# Post‐Acute Sequelae Patients with Severe COVID‐19 History Show a Prolonged Inflammatory, Vascular Injury Pattern

**DOI:** 10.1002/eji.70169

**Published:** 2026-03-29

**Authors:** Louisa Ruhl, Isabell Pink, Evgeny Chichelnitskiy, Nora Drick, Andrea Sauer, Lennart Boblitz, Kerstin Beushausen, Jana Keil, Anna‐Lena Ullrich, Julius Schmidt, Marius M. Hoeper, Tobias Welte, Jenny F. Kühne, Christine S. Falk

**Affiliations:** ^1^ Institute of Transplant Immunology Hannover Medical School Hannover Germany; ^2^ Department of Respiratory Medicine and Infectious Diseases Hannover Medical School Hannover Germany; ^3^ Department of Nephrology and Hypertension Hannover Medical School Hannover Germany; ^4^ German Centre for Lung Diseases (DZL) BREATH Site Hannover Germany; ^5^ German Centre for Infection Research (DZIF) TTU‐IICH, Hannover/Braunschweig Site Hannover Germany

**Keywords:** autoantibodies, endothelial injury, immune cell profile, inflammation, long**/**post‐Covid, soluble immune mediators

## Abstract

SARS‐CoV‐2 infection can lead to persistent symptoms (i.e., long/post‐COVID), especially in unvaccinated individuals. Little is known about the sustained impact of acute severe COVID‐19 on immune signatures and long/post‐COVID symptoms, often associated with cardiovascular events and pulmonary impairment. The longitudinal cohort (LC) was obtained from *N* = 46 patients with previous severe COVID‐19 and prior to SARS‐CoV‐2 vaccination, collected over 5 visits up to 12 months after discharge (*n* = 139). Long/post‐COVID status was assessed by lung function and fatigue scores. Blood was analysed regarding immune cell profiles, SARS‐CoV‐2 antibodies, and autoantigens. LC patients were compared with 39 acute severe COVID‐19 ICU patients and 28 unexposed pre‐pandemic donors (UE) and correlated with clinical parameters. LC patients exhibited long‐term decreased CD4^+^/CD8^+^ T cell ratio, differentiation from naïve to TEMRA, CD57^+^CCR7^−^ memory, and HLA‐DR^+^CD38^+^ activated CD4^+^ and CD8^+^ T cells. LC plasma profiles displayed elevated levels of markers for chronic inflammation and endothelial injury. Chronic inflammatory chemokines and cardiovascular markers remained high. These markers and autoantibodies against centromere structures negatively correlated with lung function. At 1‐year post‐discharge, LC patients with a COVID‐19 ICU history displayed sustained significant changes in their immune profile at cellular and inflammatory levels, revealing signatures of persistent inflammation and endothelial injury.

## Graphical abstract

Long‐COVID or post‐acute sequelae of COVID‐19 syndrome (PASC) patients with previous severe COVID‐19 ICU history prior vaccination display sustained significant changes in their immune profile at both the cellular and the inflammatory level, revealing signatures of persistent inflammation and endothelial injury.

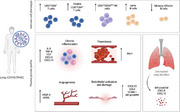

**Abbreviations**
ADI: obesity
AHT: arterial hypertension
AIC: antibody inhibition capacity
CV: cardiovascular
CVD: cardiovascular diseases
DAD: days after hospital discharge
DM: diabetes mellitus
HMVEC‐L: human lung microvascular endothelial cells
ICU: intensive care unit
LC: longitudinal COVID‐cohort
PASC: post‐acute COVID‐19 syndrome
SAEC: small airway epithelial cells
SMW: six‐minute‐walk‐test
Sub: subgroup
UE: unexposed
V: visit
VOC: variant of concern

## 1 Introduction

Long‐term effects of COVID‐19 are described with a variety of persistent symptoms, such as long‐COVID or post‐acute sequelae of COVID‐19 syndrome (PASC), and can affect several organ systems. PASC is clinically defined as persistence of symptoms or development of sequelae beyond 3 to 4 weeks after acute infection [1] and can be subdivided based on duration and composition of symptoms. Subacute COVID‐19 is characterised by persisting symptoms 4 to 12 weeks after the acute infection, whereas chronic‐/post‐COVID is determined by persisting symptoms beyond 12 weeks. PASC patients suffer from a wide range of symptoms, most commonly dyspnoea, cough, chest pain, and chronic fatigue and thromboembolic events [1]. It has been observed initially in unvaccinated patients with former severe COVID‐19, as well as in individuals with an initial mild disease course [2, 3]. The prevalence of PASC is hard to determine and depends on different factors like age, sex, vaccination status, and variant of concern (VOC) [3, 4, 5]. Frequencies for subacute COVID‐19 range from 14.5% to 18.1% and chronic‐/post‐COVID from 7.8% to 17% [4]. Increasing age, female sex, white ethnicity, poor pre‐pandemic general health, obesity, and asthma were identified as risk factors for developing PASC [4]. The underlying processes causing PASC are still under investigation, but multiple factors are discussed, including immune dysregulation, autoimmunity, tissue damage, microbiome, and persistent viral antigens [6, 7, 8, 9, 10]. In a recent cross‐sectional study by Klein et al. [11], substantial differences in myeloid and lymphoid cells, together with lower cortisol levels, were detected in LC patients versus convalescent individuals without PASC. In an epidemiological study, COVID‐19 patients were shown to be at increased risk of various cardiovascular diseases, which was also observed in non‐hospitalised COVID‐19 patients but increased in a gradient towards hospitalised and ICU patients during acute infection [12]. In our previous study on unvaccinated COVID‐19 ICU patients in 2020, we could demonstrate severe lymphopenia with expansion of hyper‐activated memory T and NK cells, a highly pro‐inflammatory plasma cytokine/chemokine milieu with core signatures associated with survival and recovery, as well as a strong impact of endothelial dysfunction as a clinically relevant part of the immunopathology [13]. Especially in these unvaccinated former COVID‐19 ICU patients, little is known about the sustained impact of severe COVID‐19 on the immune status and manifestation of PASC. Therefore, we aimed, in a longitudinal follow‐up study, to identify a potential link between immune signatures, endothelial injury, and PASC in patients with a severe COVID‐19 history. Consecutive blood samples of these patients at five visits up to 1 year after acute infection were analysed for cellular and plasma cytokine and vascular profiles, antibodies against spike‐ and N‐antigens as well as autoantigens together with lung function and fatigue parameters. By comparing the immune signature of these patients to pre‐pandemic unexposed (UE) donors and acute COVID‐19 ICU patients, we identified unique cellular and plasma protein patterns in LC patients, which also correlated with clinical PASC parameters. Especially, the plasma protein profile revealed continued inflammation, endothelial dysregulation, potentially associated with cardiovascular complications.

## 2 Results

### 2.1 Humoral Response to SARS‐CoV‐2 and Prevalence of Autoantibodies

**FIGURE 1:**
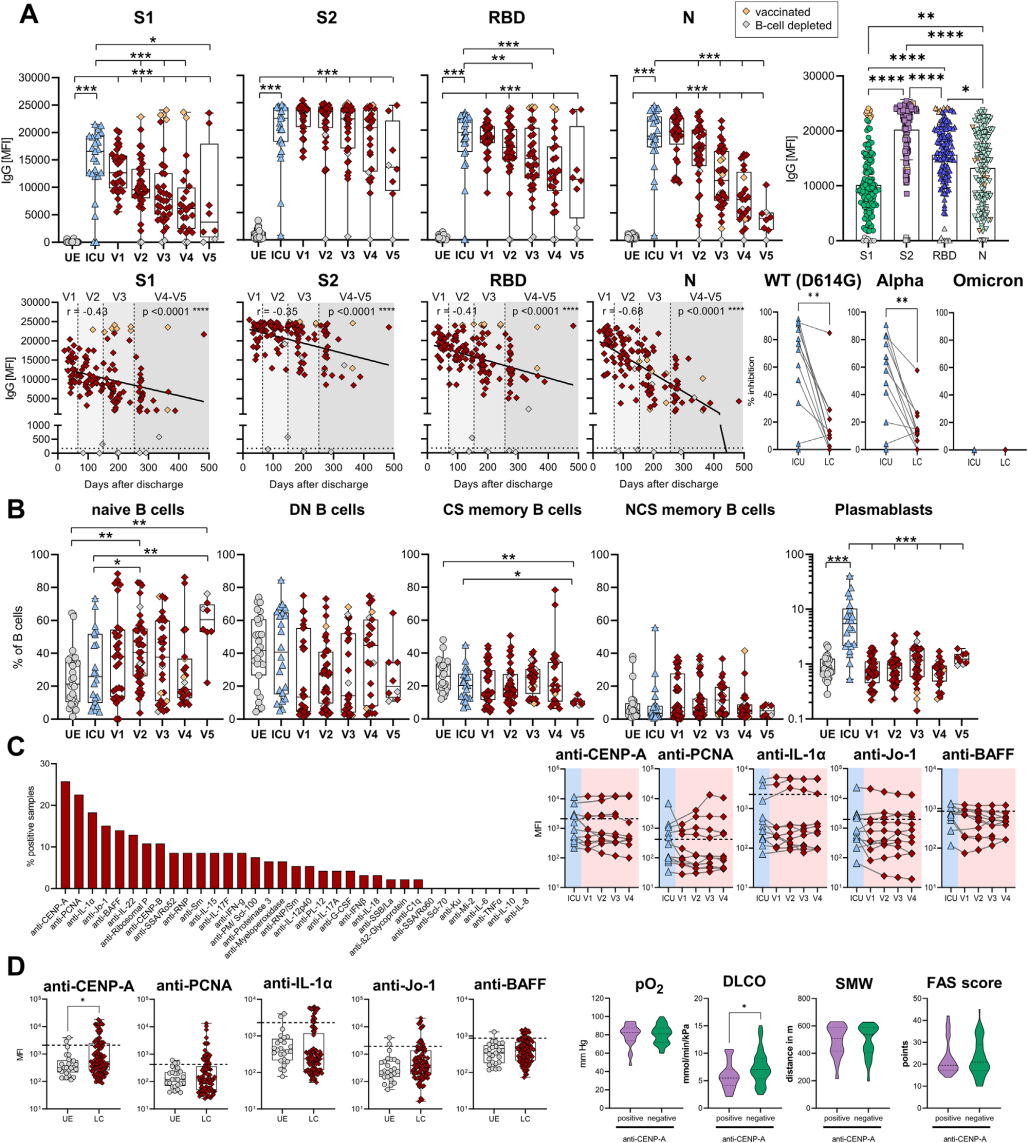
Prevalence of SARS‐CoV‐2‐specific antibodies and autoantibodies in PASC patients. **(A)** IgG antibody levels (MFI) against SARS‐CoV‐2 S1‐, S2‐domain, RBD and N‐antigen were measured with Luminex‐based multiplex assays in plasma samples from UE (*n* = 28), ICU (*n* = 25, last sample before discharge) and LC (*n* = 139, samples derived from 5 visits [V1–V5]). Spearman correlation analysis between IgG antibody levels of respective SARS‐CoV‐2 antigens from LC (*n* = 139) and days after discharge with linear regression. B‐cell‐depleted patients are highlighted in light‐grey. Vaccinated individuals are highlighted in yellow. IgG levels against S1‐, S2‐domain, RBD and N‐antigen from LC (*n* = 139). Antibody inhibitory capacity (AIC) against the spike‐protein of several SARS‐CoV‐2 variants was analysed using electrochemiluminescence‐based multiplex assays and is displayed as % inhibition. AIC against WT and VOCs (Alpha, Omicron BA.1) compared between matched samples from acute COVID‐19 ICU patients, who subsequently developed PASC (*n* = 12). Samples from the LC were collected 6 months after hospital discharge. **(B)** B‐cell subset frequencies (as % of B cells) from UE (*n* = 25), ICU (*n* = 25, last visit) and LC (*n* = 139, V1–V5) blood samples analysed by flow cytometry. B cell subsets: naive (IgD^+^CD27^−^), double negative (DN, IgD^−^CD27^−^), class‐switched memory (CS memory, IgD^−^CD27^+^), nonclass‐switched memory (IgD^+^CD27^+^) and Plasmablasts (CD19^−^CD27^+^CD38^+^). Representative gating strategy is shown in Figure S2A. B‐cell‐depleted patients are highlighted in light‐grey. Vaccinated individuals are highlighted in yellow. **(C)** Prevalence of anti‐nuclear and anti‐cytokine autoantibodies from LC plasma samples (*n* = 93) using Luminex‐based multiplex assays. Cut‐Off values were calculated based on samples from unexposed donors (*n* = 25) as the median plus double standard deviation. Data are displayed as % positive samples. Time course of autoantibody levels (MFI) in matched samples from acute COVID‐19 ICU patients (blue), who subsequently developed PASC (red, *n* = 11). **(D)** Levels of the five most prevalent autoantibodies (MFI) in LC (*n* = 93) and UE (*n* = 25) plasma samples. Clinical parameters for PASC in longitudinal‐COVID patients with (positive) or without (negative) anti‐CENP‐A autoantibodies. Autoantibodies were measured by Luminex‐based multiplex assay. DLCO: diffusing capacity of the lungs for carbon monoxide, FAS score: fatigue score, ICU: COVID‐19 ICU patients, LC: longitudinal COVID‐cohort, pO_2_: partial pressure of oxygen, SMW: six‐minute walking test, UE: unexposed donors. Statistical analysis: (A, B) Multigroup comparisons using Aligned Rank Transform ANOVA (ARTool) with FDR‐adjusted post hoc testing for non‐parametric data, or linear mixed‐effects models with Kenward–Roger correction and Tukey's post hoc test for parametric data. To account for repeated measurements, patient ID was included as a random effect. (C) Mixed effect model with Dunnett's multiple comparison test. (D) Linear mixed‐effects models with Kenward–Roger correction. *p < 0.05, **p < 0.01, ***p < 0.001.

The clinical assessment of the LC, especially at the first two visits, uncovered their impaired lung function with reduced SMW distances, pO_2_, and higher FAS score compared with non‐convalescent individuals [14]. To analyse the immunophenotype of PASC, we included 46 patients with a severe COVID‐19 ICU history as a longitudinal cohort (LC). Blood samples (*n* = 139) were taken at multiple time points, that is, V1 (6–8 weeks posthospital discharge, *n* = 32), V2 (3 months, *n* = 38), V3 (6 months, *n* = 35), V4 (9 months, *n* = 25) and V5 (12 months, *n* = 9) (Table 1; Figure S1A). All LC patients were unvaccinated in their primary acute infection; only seven patients received a SARS‐CoV‐2 vaccination after their infection. First, humoral immune responses against SARS‐CoV‐2 were determined as relative IgG levels against spike S1‐, RBD‐, S2‐domains, and N‐antigen in all LC plasma samples (*n* = 139) and partially matched acute infected ICU patients (*n* = 25 samples). Pre‐pandemic, unexposed (UE) donors (*n* = 28 samples) served as controls. LC patients displayed high IgG levels against SARS‐CoV‐2 spike and N proteins (Figure 1A) as expected. At 1‐year post‐discharge (V5), S1‐, RBD‐, S2‐, and N‐specific IgG persisted, albeit declining over time. Of note, two B‐cell‐depleted patients did not show seroconversion. Spike vaccination postinfection resulted in boosted levels of spike‐ but not N‐specific IgG. LC patients showed the highest S2‐specific IgG levels, followed by anti‐RBD, anti‐N, and anti‐S1. These results indicate long‐lasting spike‐ and N‐specific IgG in ICU‐recovered LC patients. Antibody interference assays assess the surrogate neutralising capacity of S1‐specific antibodies by blocking the binding of soluble ACE‐2 to SARS‐CoV‐2 WT or variant spike proteins, here, referred to as antibody inhibition capacity (AIC). Paired samples of *n* = 12 acute ICU and LC patients 6 months post‐discharge show a significantly decreased AIC in LC patients against WT spike, the Alpha, and most other variants. In contrast, AIC against Omicron BA.1 was 0% for ICU and paired LC samples since these patients had been infected with the WT virus, demonstrating that RBD‐specific antibodies against WT and other VOC were unable to bind with the Omicron‐specific RBD (Figure 1A lower panel; Figure S1B).

**TABLE 1:** Patient characteristics.

Characteristics	LC cohort	ICU cohort	UE cohort^a^
Number of patients, N (% of total) Number of patients with multiple samples Total sample number, n	46 (40.7%) 46 139	39 (34.5%) 25 94	28 (24.8%) 0 28
Age (mean, min, max)	55 (18.9–81.8)	56 (5.4–84.2)	46,5 (21.5–80.6)
Gender			
Male	109 (78.4%)	77 (81.9%)	12 (42.9%)
Female	30 (21.6%)	17 (18.1%)	16 (57.1%)
Outpatient clinical visits			
Visit 1 (6–8 weeks after discharge)	32	N/A	N/A
Visit 2 (3 months after discharge)	38	N/A	N/A
Visit 3 (6 months after discharge)	35	N/A	N/A
Visit 4 (9 months after discharge)	25	N/A	N/A
Visit 5 (12 months after discharge)	9	N/A	N/A
Days after hospital discharge (DAD)	159 (22–483)	N/A	N/A
Days after symptom onset (DASO)	N/A	27 (4–70)	N/A
COVID risk factors/comorbidities			
None	10		10
Obesity	17	33	5
Cardiovascular disease	12	8	1
Diabetes	12	14	5
Hypertension	21	28	7
Liver disease	6	0	0
Renal insufficiency	4	4	0
Lung disease	1	3	0
Clinical parameter			
pO_2_ (mmHg, min, max)	80.2 (24.4–52)		N/A
DLCO (mmol/min/kPa)	7.03 (2.03–15.04)		N/A
SMW (distance m, min, max)	484.46 (215–631)		N/A
FAS score (min, max)	23.01 (10–45)		N/A

*Abbreviations*: LC: longitudinal COVID‐cohort; ICU: intensive care unit patients; UE: unexposed donors; *N*: number of patients; *n*: number of samples.

^a^For reference, the unexposed control cohort as described in Ruhl et al. [13] was used.

**FIGURE 2:**
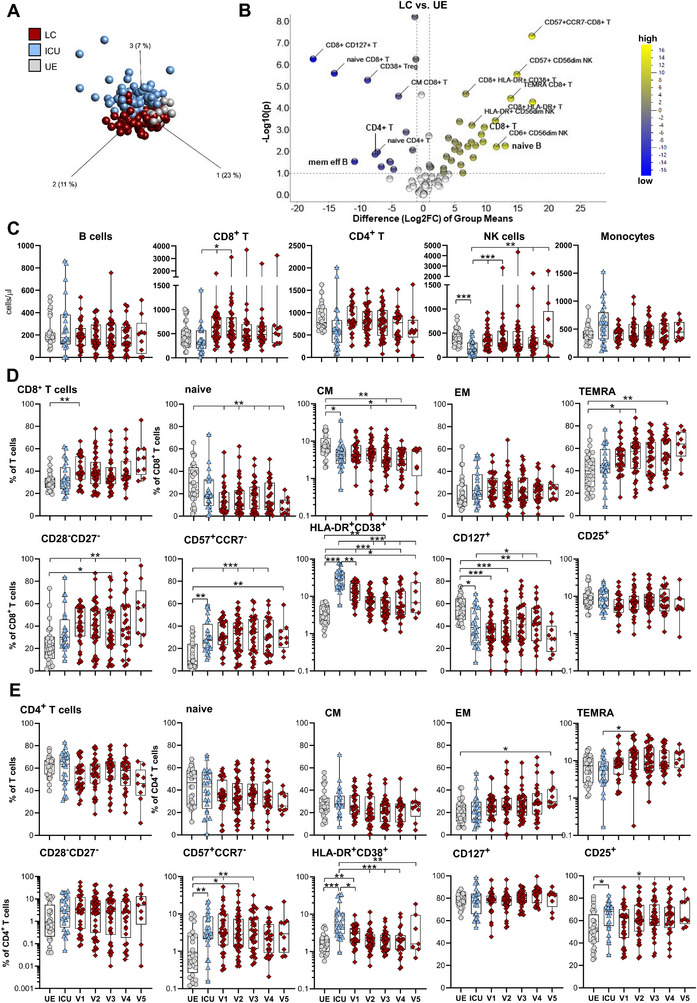
Altered immune cell phenotype of PASC patients. Immune cell composition was analysed by flow cytometry. **(A)** Principal component analysis with a multigroup comparison of blood samples for UE (*n* = 28), ICU (*n* = 94) and LC (*n* = 139, V1–V5) and a *p*‐value cut‐off of 0.004 was used to identify significantly different immune subsets between the three patient cohorts. **(B)** Volcano plot visualising two‐group comparison of immune cell frequencies from UE (*n* = 28) and LC (n = 139, V1–V5). **(C)** Absolute cell numbers (cells/µL) of major immune cell populations in patient blood were quantified using TruCount analyses for UE (*n* = 28), ICU (*n* = 25) and LC (*n* = 139, V1–V5). Representative gating strategy is shown in Figure S2B. **(D, E)** Immune cell frequencies from UE (*n* = 28), ICU (*n* = 25, last visit before discharge) and LC (*n* = 139, V1–V5) for CD8^+^ T cell subsets in **(D)** and CD4^+^ T cell subsets in **(E)**. T cells: naive (CCR7^+^CD45RO^−^), central memory (CM, CCR7^+^CD45RO^+^), effector memory (EM, CCR7^−^CD45RO^+^) and TEMRA (CCR7^−^CD45RO^−^). Representative gating strategy is shown in Figure S2C. ICU: COVID‐19 ICU patients, LC: longitudinal COVID‐cohort, UE: unexposed donors. Statistical analysis: (C, D) Multigroup comparisons were performed using Aligned Rank Transform ANOVA (ARTool) with FDR‐adjusted post hoc testing for non‐parametric data, or linear mixed‐effects models with Kenward–Roger correction and Tukey's post hoc test for parametric data. To account for repeated measurements, patient ID was included as a random effect. *p < 0.05, **p < 0.01, ***p < 0.001.

The B cell phenotype showed an increase in IgD^+^CD27^−^ naïve B cells in ICU but also in LC patients (Figure 1B; Figure 2B). Simultaneously, proportions of IgD^−^CD27^−^ double‐negative B cells declined. Frequencies of IgD^−^CD27^+^ class‐switched (CS) memory, IgD^+^CD27^+^ nonclass‐switched (NCS) memory B cells, and CD27^+^CD38^+^ plasmablasts were stable in LC patients at a lower level compared with ICU samples. These results indicate sustained de novo B cell development after acute infection.

Other studies reported the occurrence of autoantibodies to be associated with severe COVID‐19 and the manifestation of PASC [15]. Therefore, we analysed the prevalence of anti‐nuclear and anti‐cytokine autoantibodies in LC (*n* = 93) and paired ICU (*n* = 11) samples. Cut‐off values were calculated based on unexposed donor samples (*n* = 25) as the median MFI plus double standard deviation. Despite lacking blood samples before SARS‐CoV‐2 infection in these patients, CENP‐A autoantibodies were most prevalent with 25% of LC samples, followed by PCNA, IL‐1α, Jo‐1, and BAFF (Figure 1C). Paired follow‐up samples from *N* = 11 ICU patients showed stable autoantibody levels over 9 months post‐discharge (Figure 1C). Autoantibodies targeting inflammatory cytokines like IL‐6, TNF‐α, and CXCL8/IL‐8 were not detected in this small cohort. Overall, the occurrence of autoantibodies was highly variable. Only the proportion of CENP‐A autoantibody‐positive samples was significantly increased in LC patients compared with pre‐pandemic unexposed (UE) donors (Figure 1D). Interestingly, lung function evaluated by diffusing capacity of the lungs for carbon monoxide (DLCO) was significantly reduced in LC patients with anti‐CENP‐A autoantibodies (Figure 1D). However, partial pressure of oxygen (pO_2_), six‐minute walking test (SMW), and fatigue (FAS) score were unaltered in LC patients with or w/o anti‐CENP‐A autoantibodies. Also, the other most prevalent autoantibodies specific for PCNA, IL‐1α, Jo‐1, or BAFF were not linked to these clinical parameters (Figure S3). Hence, a general link between the presence of autoantibodies and the manifestation of LC could not be made, taking the small study size into account.

### 2.2 Immune Cell Phenotype Indicates Sustained Memory T Cell Development

The leukocyte composition of these LC patients (*n* = 139 samples in total, derived from five visits [V1–V5]) was investigated via flow cytometry, focusing on T, B, and NK cell subsets. The same ICU patients (*n* = 25, last samples before discharge) and UE donors (*n* = 28) served as controls. PCA and volcano plot analyses with 96 immune cell populations clearly separated LC from both ICU patients and UE donors (Figure 2A,B). Total T and NK cell numbers recovered in LC patients at V1 to V5 from their lymphopenic status during ICU, while B cells, monocytes, and granulocytes remained stable (Figure 2B,C; Figure S4). Compared with pre‐pandemic unexposed (UE) donors, LC patients still showed trends towards reduced CD4^+^ and increased CD8^+^ T cell frequencies that did not reach statistical significance (Figure 2D,E). Moreover, LC patients were characterised by a sustained memory T and NK cell phenotype (Figure 2B,D,E; Figure S2C), described by decreased frequencies of CCR7^+^CD45RO^−^ naïve and concurrently increased proportions of CCR7^−^CD45RO^−^ TEMRA CD4^+^ and CD8^+^ T cells, as could already be seen during the acute disease requiring ICU hospitalisation. An activated and terminally differentiated memory phenotype was particularly pronounced in the CD8^+^ T cell compartment with enlarged proportions of CD28^−^CD27^−^ memory, CD57^+^CCR^−^ late memory, and HLA‐DR^+^CD38^+^ activated CD8^+^ T cells. CD4^+^ T and NK cells also showed higher activation levels, with elevated HLA‐DR^+^CD38^+^ CD4^+^ T and HLA‐DR^+^CD56^dim^ NK cell frequencies (Figure 2D; Figure S5A), which slowly declined. CD4^+^CD25^high^CD127^low^ regulatory T cells (Treg) were only slightly increased and stable compared with both UE and ICU samples (Figure S5B,C). These findings reveal a continuously altered immune cell phenotype in LC/PASC patients characterised by activated and terminally differentiated memory T, primarily CD8^+^ T, and NK cells.

### 2.3 Correlates of the Immune Cell Phenotype With Age and Clinical Long/Post‐COVID Features

**FIGURE 3:**
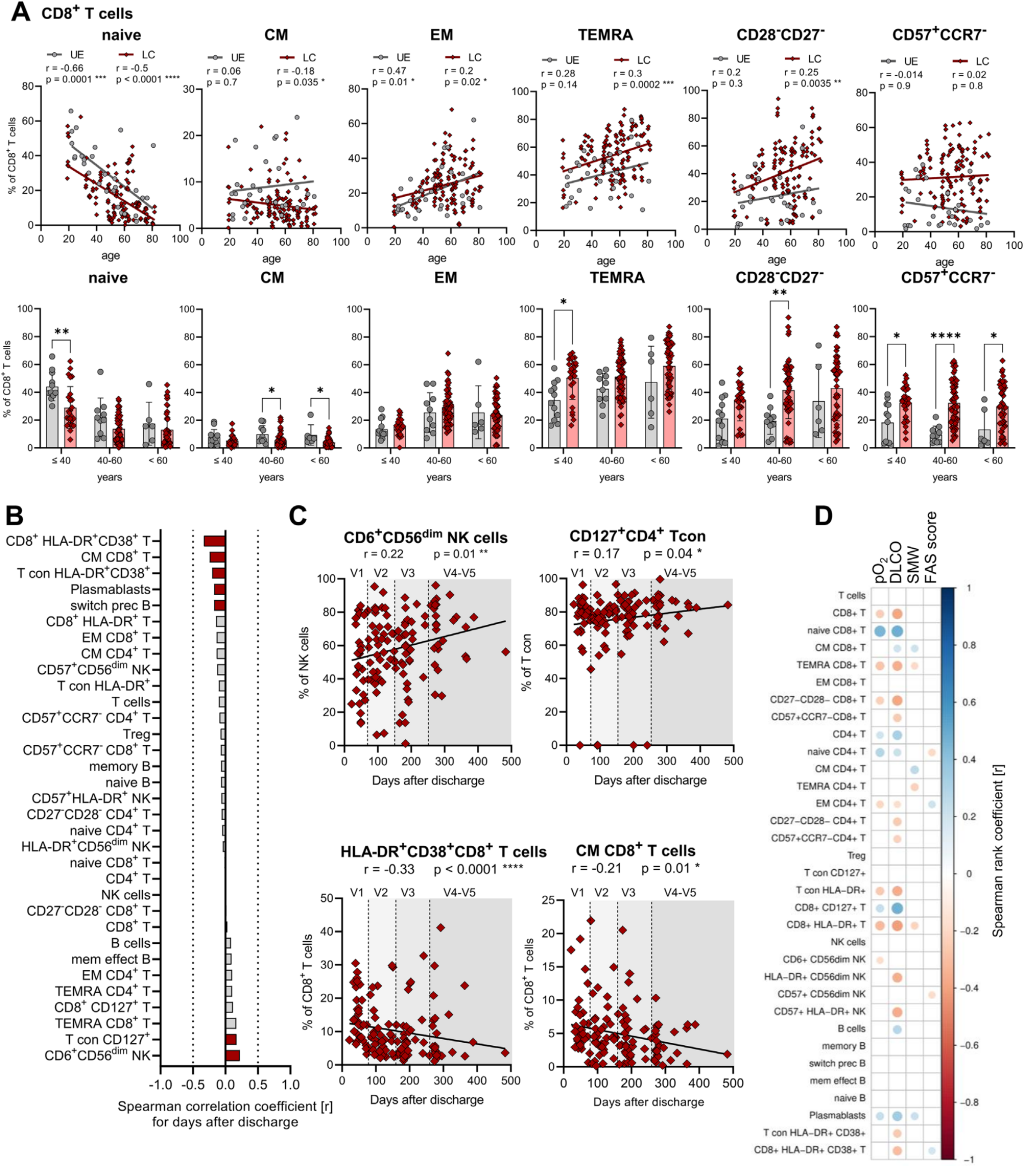
Immune cell phenotype changes correlate with clinical symptoms of PASC. Immune cell composition was analysed by flow cytometry. **(A)** CD8^+^ T cell subset frequencies (as % of CD8^+^ T cells) in different age groups of LC and UE. UE are displayed in grey, LC are displayed in red. UE: ≤40 years, *n* = 12; 40–60 years, *n* = 9; >60 years, *n* = 7; LC: ≤40 years, *n* = 25; 40–60 years, *n* = 54; >60 years *n* = 60. Spearman correlation analysis between proportions of different T cell subsets and age from LC (*n* = 139) and UE (*n* = 25) with linear regression. **(B)** Waterfall plot representing the correlation coefficient (*r*) of Spearman correlation analysis between proportions of the most relevant immune cells and days after hospital discharge (DAD) from LC (*n* = 139). Red columns represent significant results. **(C)** Spearman correlation analysis between proportions of different immune cell subsets from LC (*n* = 139) and days after discharge with linear regression. **(D)** Correlation matrix of the most relevant immune cell subsets correlated with clinical parameters for PASC: pO2, DLCO, SMW, and FAS score. Spearman correlation was used to calculate correlation coefficients, which were displayed in circles. The red to blue scale represents the correlation coefficient (*r*). DLCO: diffusing capacity of the lungs for carbon monoxide, FAS score: fatigue score, ICU: intensive care unit patients, LC: longitudinal COVID‐cohort, pO_2_: partial pressure of oxygen, SMW: six‐minute walking test, UE: unexposed donors. Statistical analysis: (A, C) Spearman rank correlation, (A) two‐way ANOVA. *p < 0.05, **p < 0.01.

Age significantly impacts immune cell composition, particularly memory T cell differentiation. Since we observed a loss of naïve and gain of memory CD4^+^ and CD8^+^ T cells in LC patients (Figure 2D,E), we analysed whether this was associated with an increased patient age. However, correlation analyses revealed that LC patients consistently showed lower proportions of naïve and higher proportions of memory T cells compared with UE donors (Figure 3A; Figure S6A). This effect was more pronounced in younger LC patients (≤40 years), who exhibited significantly reduced naïve CD8^+^ and CD4^+^ T cells compared with age‐matched unexposed controls (Figure 3A; Figure S6B). Due to lymphopenia in acute COVID‐19 ICU patients, leading to the disruption of the age correlation otherwise observed in healthy individuals, this particular patient cohort was not included in this analysis.

Younger LC patients also displayed significantly elevated levels of TEMRA CD8^+^ T cells, indicating stronger and sustained T cell differentiation. Notably, late memory CD57^+^CCR7^−^ CD8^+^ T cell frequencies were increased in all age groups of LC patients. These results suggest a shift in the T cell phenotype of LC/PASC patients towards a memory phenotype, especially in younger individuals. Next, correlation analyses with 33 immune cell subsets and days after hospital discharge (DAD) ranging from 22 to 483 days, were performed to test the persistence of immune cell phenotype alterations within 1‐year post‐hospital discharge. Out of 33, 26 immune cell populations were consistently altered within this timeframe, including various memory T cell subsets, indicating the differentiation of memory T cells (Figure 3B). Only a few immune cell subsets changed over time, such as highly differentiated late memory CD57^+^CD56^dim^ NK cells, which increased within 1 year after COVID‐19 infection. Proportions of highly activated T cells represented by HLA‐DR^+^CD38^+^ CD4^+^ Tcon and CD8^+^ T cells decreased over time, that is, V1–V5 (Figure 3B,C). These results imply a consistent and long‐lasting memory phenotype of T and NK cells in LC patients within 1 year after acute infection, also in younger individuals. The impact of immune cells on PASC scores was assessed by correlating these 33 immune cell subsets with four clinical PASC‐associated parameters, that is, pO_2_, DLCO, SMW, and FAS score (Figure 3D). Remarkably, CD8^+^ T cells, particularly activated HLA‐DR^+^, differentiated TEMRA and memory CD27^−^CD28^−^CD8^+^ T cells negatively correlated with DLCO and pO_2_, implying a link to reduced pulmonary function. Conversely, proportions of naïve CD8^+^ and CD4^+^ T cells, as well as CD4^+^ T cells in general, were positively correlated with pO_2_ and DLCO. Interestingly, none of the analysed immune cell subsets correlated with the FAS score (Figure 3D). Thus, decreased lung function in LC patients may be associated with CD8^+^ T cell activation and differentiation. However, further studies (i.e., bigger sample size) are needed before definitive conclusions can be drawn.

### 2.4 Distinct Plasma Protein Signatures in LC Versus ICU Patients and UE Donors

**FIGURE 4:**
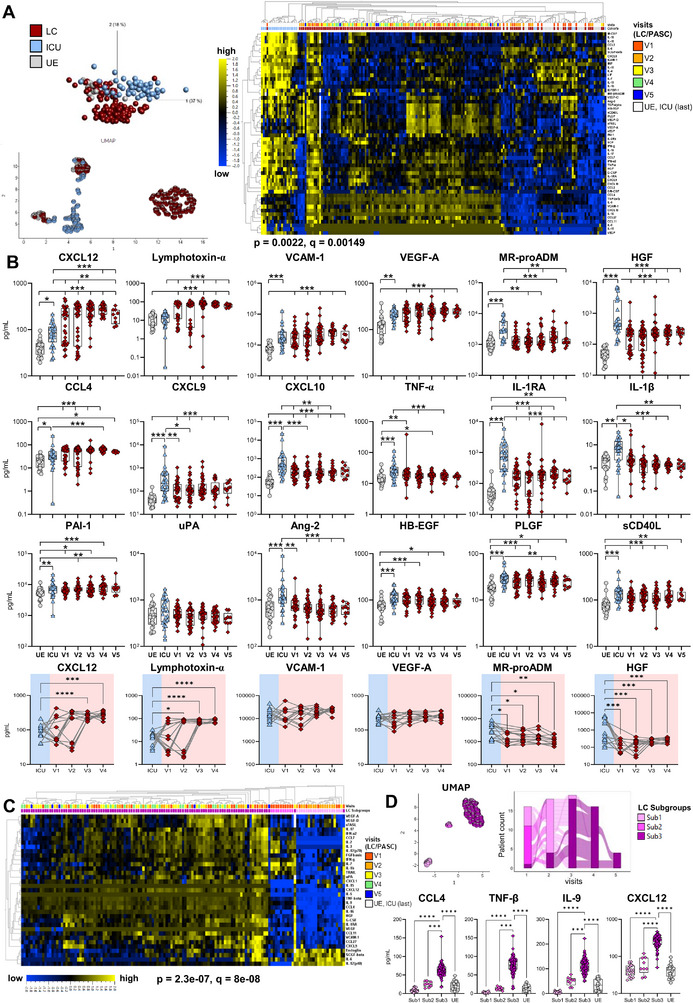
Altered plasma protein signature of PASC patients. Plasma protein concentrations in patient plasma were measured by a Luminex‐based multiplex assay. **(A)** Multigroup comparison with a *p*‐value cut‐off of 0.002, FDR <0.05 was used to identify significant differences between UE (*n* = 26, grey), ICU (*n* = 25, last visit before discharge, light blue) and LC (*n* = 139, V1–V5, dark red) for principal component analysis, UMAP and unsupervised hierarchical clustering (heatmap) analyses. For LC samples, the heatmap includes colour coding per visit (V1 – light red, V2 – orange, V3 – yellow, V4 – green, and V5 – cobalt blue). Blue to yellow scale represents the z‐score normalised concentrations normalised to mean = 0, var = 1. *p*‐values were adjusted for multiple testing using the Benjamini–Hochberg method. Missing values are displayed in white. **(B)** Plasma protein concentrations (in pg/mL) from UE (*n* = 26), ICU (*n* = 25, last visit before discharge), and LC (n = 139, V1‐V5) samples. Time course of plasma protein levels (pg/mL) in matched samples from acute COVID‐19 ICU patients (blue), who subsequently developed PASC (red, *n* = 11). **(C)** Heatmap analysis of plasma proteins from LC (*n* = 139), as in A, including colour coding according to LC subgroups 1–3. Blue to yellow scale represents the expression values normalised to mean = 0, var = 1. Missing values are displayed in white. **(D)** UMAP clustering of plasma proteins for LC (*n* = 139) according to LC subgroups 1–3. Alluvial plot analysis of N = 19 LC according to LC subgroups 1–3. Plasma protein levels of CCL4, TNF‐β, IL‐9 and CXCL12 (in pg/mL) in UE (*n* = 26) and LC subgroups (Sub1: *n* = 20, Sub2: *n* = 10, Sub3: status *n* = 109). Statistical analysis: (B, D) Multigroup comparisons were performed using Aligned Rank Transform ANOVA (ARTool) with FDR‐adjusted post hoc testing for non‐parametric data, or linear mixed‐effects models with Kenward–Roger correction and Tukey's post hoc test for parametric data. To account for repeated measurements, patient ID was included as a random effect. *p < 0.05, **p < 0.01, ***p < 0.001.

Next, we analysed the plasma protein signatures of 46 LC patients (*n* = 139 samples from five visits (V1: *n* = 32, V2: *n* = 38, V3: *n* = 35, V4: *n* = 25, and V5: *n* = 9) and compared them to partially paired COVID‐19 ICU patients (*n* = 25, last ICU samples) and pre‐pandemic unexposed (UE) donors (*n* = 26). In our previous publication, we already identified a protein signature associated with hyperinflammation, endothelial disruption, and dysregulated coagulation in COVID‐19 ICU patients [13]. Unsupervised clustering of 66 plasma proteins showed a clear distinction between LC, acute ICU patients, and UE donors, with 59 proteins significantly differing between the groups (Figure 4A). LC/PASC patients exhibited an altered plasma protein signature, with even continuously higher levels of CXCL12, CCL4, IL‐9, lymphotoxin‐α, and sFASL than in the ICU during active disease. Similarly, VCAM‐1, VEGF‐A, VEGF‐D, PAI‐1, CCL27, and G‐CSF levels were higher in LC and ICU patients compared with UE (Figure 4A,B; Figure S7). Together with increased levels of other soluble mediators like CXCL9, CXCL10, TNF‐α, HGF, and sCD40L, this further indicates continuous inflammation and endothelial dysregulation from V1 to V5 in LC/PASC patients (Figure 4B). MR‐proADM, a potential biomarker for vascular dysfunction and cardiovascular (CV) complications, was also still significantly increased in LC patients. Thus, the increase in the endothelial activator TNF‐α and the CV injury marker MR‐proADM pointed to persistent vascular injury in LC patients, which was also associated with acute COVID‐19 severity [13]. Yet, clinical parameters, that is, NT‐proBNP levels, for LC/PASC participants with known CV complications were within normal range.

In contrast, uPA, Ang‐2, IL‐1β, IFN‐γ, and IL‐6, which were elevated in acute COVID‐19 ICU patients, returned to baseline levels in LC patients (Figure 4B; Figure S7). Longitudinal paired follow‐up samples from COVID‐19 ICU patients (*N* = 14) confirmed increasing levels of CXCL12 and lymphotoxin‐α post‐discharge, remaining high for up to 9 months (V4) in some patients (Figure 4B bottom panel). Taken together, the plasma profile of LC patients differed significantly from both ICU and UE samples, indicating continuous endothelial injury and chronic inflammation.

**FIGURE 5:**
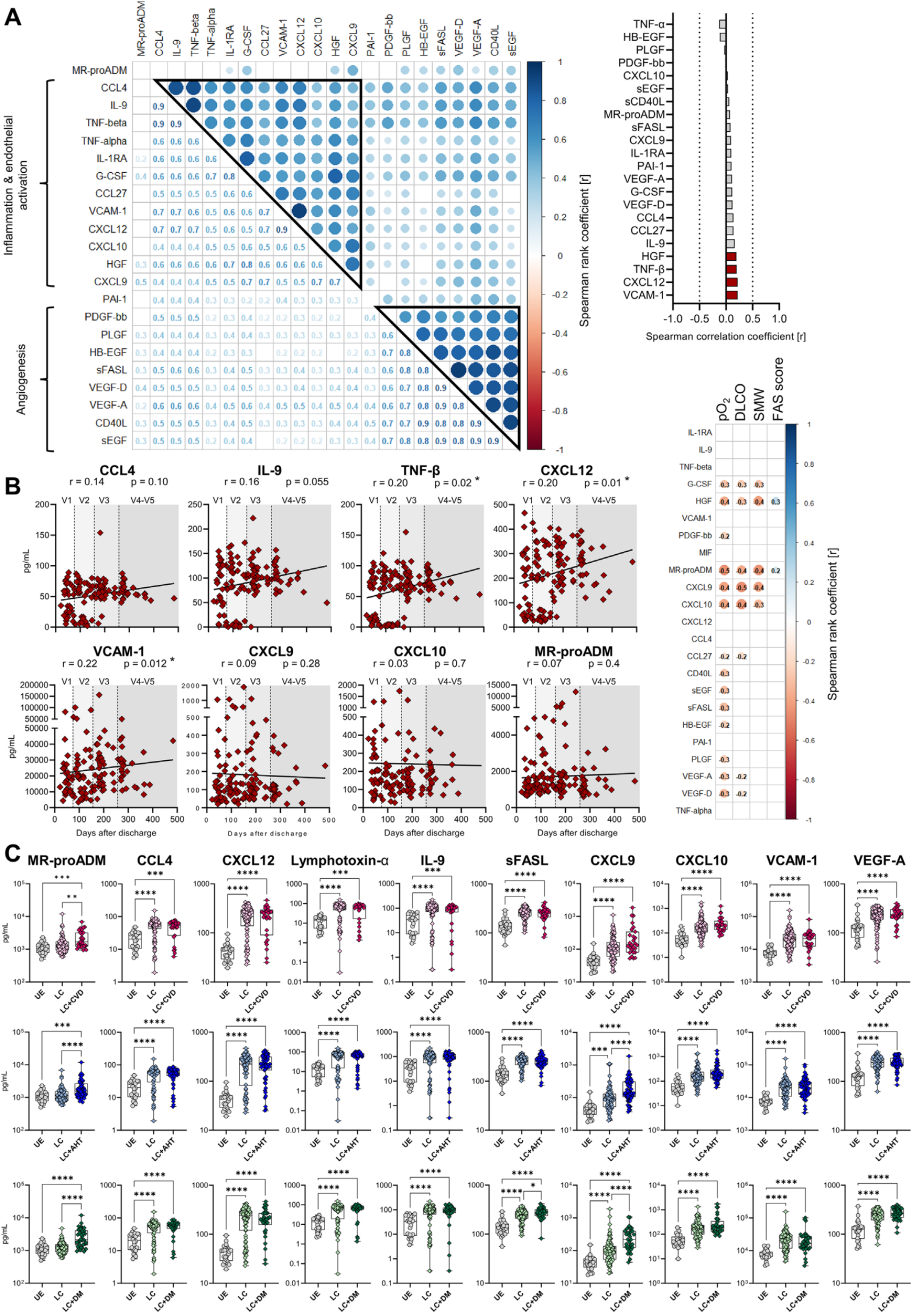
Altered plasma protein signature correlated with PASC symptoms. Plasma protein concentrations in patient plasma were measured by a Luminex‐based multiplex assay. **(A)** Mixed correlation matrix of the most relevant plasma proteins from LC (*n* = 139). Spearman correlation was used to calculate correlation coefficients, which are displayed in circles on the upper right side. The significance of correlation is depicted by circle size, while the strength and direction of the correlation are displayed by colour code. Plasma proteins were ordered using hierarchical clustering. The red to blue scale represents the correlation coefficient (*r*). Concrete numbers of Spearman's rank correlation coefficient (*r*) are displayed at the lower left side. Waterfall plot representing the correlation coefficient (*r*) of Spearman correlation analysis between concentrations of most relevant plasma proteins and days after hospital discharge (DAD) from LC (*n* = 139). Red columns represent significant results. Dotted lines represent the Spearman correlation coefficient (*r*) of 0.5 and −0.5. **(B)** Spearman correlation analysis between concentrations of selected plasma proteins and DAD from LC (*n* = 139, V1–V5) with linear regression. Correlation matrix of the most relevant plasma proteins with clinical parameters for PASC: pO_2_, DLCO, SMW, and FAS score. Spearman correlation was used to calculate correlation coefficients, which were displayed in circles. The red to blue scale represents the correlation coefficient (*r*). Concrete numbers of Spearman's rank correlation coefficient (*r*) are displayed within the circles. **(C)** Plasma protein levels in UE and LC. LC/PASC were subdivided into longitudinal PASC patients without underlying disease and longitudinal PASC patients with a certain underlying disease (i.e. cardiovascular disease (CVD), arterial hypertension (AHT), diabetes mellitus (DM). DLCO: Diffusing capacity of the lungs for carbon monoxide, FAS score: fatigue score, pO_2_: partial pressure of oxygen, SMW: six‐minute walking test. Statistical analysis: (B) Spearman rank correlation, (C) mixed model test corrected for repeated measurements with Dunn's multiple comparison test. *p < 0.05, **p < 0.01, ***p < 0.001, ****p < 0.0001.

Unsupervised PCA and UMAP analyses of these proteomic data revealed that LC/PASC patients could be divided into three subgroups (Figure 4C,D). Subgroup (Sub) 1 (*n* = 20) and Sub2 (*n* = 10) exhibited a plasma protein profile similar to UE donors, while Subgroup 3 (*n* = 109) displayed elevated levels of CCL4, lymphotoxin‐α, IL‐9, CXCL12, CXCL9, CXCL10, VCAM‐1, and TNF‐α. Compared with UE donors, Sub1 displayed increased endoglin, SCGF‐b, IL‐6, and IL‐12(p40) levels (Figure 4C,D; Figure S8A), which were also shown to be elevated in acute COVID‐19 ICU patients [13]. Interestingly, longitudinal follow‐up of 19 patients in the alluvial plot showed that the subgroup assignment changed from V1 to V4 finally with all patients belonging to Sub3 (Figure 4D). Hence, plasma protein profiles seem to undergo a stepwise development with increasing levels of CXCL12, CCL4, TNF‐𝛼, and IL‐9. Notably, no differences in clinical parameters or underlying diseases were found for these three PASC patient subgroups, but rather the longitudinal time point after ICU release was the main driver of these profiles (Figure S8B,C). We believe that these findings and the dynamics in cytokine profiles, which are linked to the recovery after COVID‐19, may be clinically relevant. Again, further studies (i.e., increased cohort size) are needed as they will be helpful for definite conclusions.

### 2.5 Correlates of the Proteomic Profile With Time After Acute Severeinfection (ICU)

We performed further statistical analyses with the 22 most significantly increased plasma proteins, termed the core plasma signature. Correlation analysis identified two protein clusters. The first included proteins associated with angiogenesis, such as VEGF‐A, PLGF, and HB‐EGF (Figure 5A). The second cluster comprised factors associated with chronic inflammation and endothelial activation, suggesting co‐regulated secretion of these proteins, emphasising the close relationship between inflammation and endothelial activation. TNF‐α, a key driver of endothelial activation, correlated with levels of PASC‐associated proteins like CCL4, IL‐9, Lymphotoxin‐α, G‐CSF, VCAM‐1 and CXCL12, potentially arguing for an inflammation‐driven endothelial activation in LC patients. In correlation analyses between core plasma protein levels and DAD, we found that the proteomic signature remained stable within 1 year. Levels of HGF, Lymphotoxin‐α, CXCL12, and VCAM‐1 slightly increased over time while CCL4, IL‐9, CXCL9, CXCL10, PAI‐1, and MR‐proADM remained at elevated levels (Figure 5A,B). Notably, levels of MR‐proADM, CXCL9, and CXCL10 negatively correlated with clinical parameters pO_2_, DLCO, and SMW, suggesting that previous severe COVID‐19 may contribute to a connection between endothelial activation/inflammation and reduced lung function in LC/PASC patients (Figure 5B). In contrast, the fatigue score was only slightly positively correlated to HGF and MR‐proADM, with no correlation to other plasma proteins. However, enhanced levels of MR‐proADM, a marker for CV injury and vascular dysfunction, and its correlation with pulmonary impairment, may point towards a potentially increased risk for CV complications in LC/PASC patients previously suffering from severe COVID‐19.

Considering the association of severe COVID‐19 with underlying metabolic diseases, we examined the impact of underlying conditions like cardiovascular diseases (CVD), arterial hypertension (AHT), diabetes mellitus (DM), and obesity (ADI) on the plasma protein profile of LC/PASC patients. Importantly, levels of several plasma proteins were equally elevated between LC patients with and without these conditions, showing that these do not directly impact the plasma protein profile of LC patients. MR‐proADM levels were increased in LC patients with primary CVD, AHT, and DM (Figure 5C). CXCL9 and CXCL10 were increased in all LC patients but further increased in those with CVD, AHT, and DM (Figure 5C; Figure S9). Thus, the plasma protein profile changes in LC patients were primarily associated with post‐COVID pulmonary impairment but also pointed toward increased risk of metabolic diseases in these previously severe COVID‐19 patients. Yet further studies, as well as an enlarged patient cohort, are needed to draw definite conclusions on this matter.

In in vitro stimulation assays using primary human lung microvascular endothelial cells (HMVEC‐L) and small airway epithelial cells (SAEC), we could demonstrate the contribution of endothelial rather than epithelial cells to the inflammatory plasma profiles (Figure S10). Stimulation with LPS, IL‐1β, TNF‐α, IFN‐α, and IFN‐γ increased mRNA expression of VCAM1, PLAU (uPA), and *SERPINE‐1* (PAI‐1) only in HMVEC‐L. These cells secreted CCL4, CXCL12, IL‐9, Lymphotoxin‐α, CXCL9, CXCL10, VCAM‐1, and HGF, which were found to be characteristically increased in LC patients. These findings suggest that human lung endothelial cells are highly responsive to inflammatory cytokines like TNF‐α, secreting various cytokines, chemokines, and endothelial factors. Thus, plasma protein signatures in LC patients appear to be driven by both ongoing inflammation and endothelial activation. In contrast, lung epithelial cells seem to moderately contribute to inflammation and vascular dysregulation.

## 3 Discussion

In this study, patients after severe acute COVID‐19 (ICU) in the early phase of the pandemic, before vaccination with a severe SARS‐CoV‐2 WT infection, were followed in 5 visits up to 12 months regarding their immune cell composition, humoral SARS‐CoV‐2 response, autoantibodies, and plasma protein profiles, as well as clinical parameters associated with PASC. All immunocompetent patients displayed spike‐ and N‐specific IgG with declining but still detectable levels over time. Anti‐CENP‐A autoantibodies were detectable in more than 25 % of LC patients and associated with impaired lung function. These autoantibodies are usually seen in systemic sclerosis (SSc) [16, 17], a disease characterised by fibrotic processes in the skin but also visceral organs like the heart or lungs. For instance, anti‐CENP‐A autoantibodies are known to be associated with vascular dysfunction and pulmonary complications in SSc [16, 18]. Decreased lung function in LC patients with anti‐CENP‐A autoantibodies argues for monitoring for SSc development in these individuals, since autoimmunity may be related to PASC symptoms as discussed by several other publications [3, 7, 8, 9, 10].

At the cellular level, PASC manifestation with decreased pO_2_ and DLCO correlated with activation and differentiation of CD8^+^ but also CD4^+^ T and NK cells, as well as inflammatory chemokines CXCL9/10, and MR‐proADM as a marker for endothelial disruption and CV complications. This combination suggests that inflammatory events may negatively affect lung function and endothelial homeostasis. CD8^+^ T cell activation has also been associated with decreased lung function in other studies. For instance, PASC patients displayed high proportions of IFN‐γ‐ and TNF‐α‐producing CD4^+^ and CD8^+^ T cells linked to reduced pulmonary function [19]. Additionally, respiratory CD8^+^ T cells were postulated to be correlated with impaired lung function [20]. Taken together, our study and those of others suggest inflammation‐driven lung pathologies in patients after COVID‐19, which may be related to CD8^+^ T cells. MR‐proADM is known as a potential biomarker for CV complications [21], and elevated plasma levels have been associated with CV risk factors and manifestations like hypertension, dyslipidaemia, CVD, and heart failure [12]. In addition, Xie et al. [12] reported an increased risk of CV events for patients following acute COVID‐19, particularly after severe courses. Thus, increased MR‐proADM levels together with elevated markers for endothelial injury may serve as indicators for potential CV complications in LC patients with previous severe COVID‐19. Yet further studies are needed to draw definite conclusions, particularly on a potential link between post‐acute COVID‐19, CV complications, and autoimmunity.

Recent studies report long‐lasting impaired lung function in patients after COVID‐19, especially hospitalised COVID‐19 patients [22, 23]. Our proteomic analysis revealed a distinct plasma protein profile, with CXCL9 and CXCL10 being associated with decreased lung function. These chemokines are also known to be elevated in sarcoidosis and autoimmune‐associated pneumonia [24]. CXCL9/10 have been further described as angiostatic factors, which implies inhibited angiogenesis in patients after COVID‐19. However, we also observed higher levels of pro‐angiogenic factors like CXCL12, VEGF‐A, VEGF‐D, and PDGF‐bb in these individuals. The simultaneous occurrence of angiostatic and pro‐angiogenic factors argues for an imbalanced vessel formation, which could drive aberrant neo‐angiogenesis in the lung. In this regard, Ackermann et al. [25] observed intussusceptive angiogenesis, which represents an unusual type of vessel formation, in deceased severe COVID‐19 patients and could also impact lung function in patients who survived COVID‐19. Remarkably, we found pro‐angiogenic factors associated with pulmonary remodelling [26], pulmonary arterial hypertension [27], and acute lung injury [28], like CXCL12, VEGF‐A, VEGF‐D, and PDGF‐bb, to remain elevated up to 1‐year post‐discharge. Since these proteins are also linked to CV events [29, 30, 31], our study points towards a link between endothelial homeostasis, angiogenesis, and PASC manifestation.

Importantly, our study focused on patients with severe ICU COVID‐19 history in the era of SARS‐CoV‐2 WT virus and before vaccination, and other studies, including our own, demonstrate that initial COVID‐19 severity affects PASC‐associated symptoms and immune profiles [14]. In contrast to our study, Klein et al. [11] enrolled PASC‐patients with primarily mild acute COVID‐19 without longitudinal follow‐up of individual patients. They found absolute counts of CD4^+^ T cells to be increased and no changes in the compartment of naïve CD4^+^ and CD8^+^ T cells, which is in contrast to our study with LC patients with severe acute COVID‐19 history in the ICU. In this elegant cross‐sectional study by Klein et al. [11], multidimensional immune phenotyping and unbiased machine learning identified biological features associated with PASC, for instance, differences in circulating myeloid and lymphocyte populations, antibodies against other viruses, lower cortisol levels, and increased inflammation. Among a variety of different immune parameters and techniques, they also reported increased CCL4 levels in correlation to LC [11]. Thus, both studies point towards an impact of the individual immune status in LC patients but also to differences based on the severity of acute infection, VOC, concomitant diseases, and status before SARS‐CoV‐2 vaccination.

### 3.1 Data Limitations and Perspectives

The limitations of our observational study include the single‐centre setting with a rather small and heterogeneous patient cohort in the era of WT virus before vaccination, and with no paired pre‐infection samples. We are well aware of the special ‘unvaccinated’ feature of the LC and ICU study population, but it provides a clear infection‐driven insight without interference of vaccine responses. Nevertheless, we believe that our findings are relevant for PASC patients independently of the vaccination status. SARS‐CoV‐2 has evolved, and VOCs have emerged, characterised by for instance reduced COVID‐19 disease severity for the Omicron VOC, resulting in fewer reported cases of LC/PASC. Despite this interplay between viral evolution, immune response, and escape features, we argue that our findings may also apply to individuals of the post‐vaccine and/or omicron era affected by LC/PASC.

Furthermore, samples of ICU patients were obtained at variable time points during the disease according to the clinical procedure in Lower Saxony, with MHH serving as a tertiary care centre for severe cases in need of advanced life support. Therefore, a prospective study design with defined time points was not feasible. Nevertheless, time‐dependent alterations could be identified by combining patient data, a procedure that has been utilised for many COVID‐19 studies. Of course, further studies are needed to define the therapeutic consequences of our observations, especially with respect to their impact on long‐COVID/PASC, but also for drawing definite conclusions on the i.e. cardiovascular complications.

In summary, our study revealed unique immune cell patterns and plasma protein profiles in a longitudinal cohort of patients after severe ICU COVID‐19, along with a higher prevalence of anti‐CENP‐A autoantibodies. These immune cell and plasma protein changes persisted over time, indicating chronic inflammation and endothelial injury that may increase the risk for underlying conditions in patients with previous severe COVID‐19. Additionally, anti‐CENP‐A autoantibodies and specific plasma proteins associated with vascular disruption, CV complications and inflammation were linked to reduced lung function in our PASC patients. Though more studies are needed to definitively conclude on a potential association between post‐acute COVID‐19, cardiovascular complications and autoimmunity.

## 4 Materials and Methods

### 4.1 Study Design and Clinical Parameters

In total, 46 convalescent, unvaccinated patients with previous severe, ICU‐treated COVID‐19 (LC) were recruited between May 2020 and September 2021 at MHH (Figure S1A). All patients hospitalised due to acute COVID‐19 were offered a follow‐up appointment at the specialised outpatient clinics of the Departments of Respiratory Medicine by MHH [14]. Blood samples and clinical data were obtained at 6 to 8 weeks (V1), 3 (V2), 6 (V3), 9 (V4), and 12 months (V5) after hospital discharge, resulting in a longitudinal paired sample collection. In longitudinal PASC patients, clinical parameters were assessed, such as pO_2_ (mmHg), SMW (6 min‐walk‐test, distance m), DLCO, FAS fatigue score, as well as comorbidities [14]. Cardiovascular diagnostics (i.e. quantification of NT‐proBNP levels) were performed in LC patients with known heart failure and were within normal levels.

Patient characteristics for control groups (COVID‐19 intensive care unit patients [ICU]) and pre‐pandemic, unexposed (UE) donors are summarised in Table 1 and have been described previously [13]. ICU patient sera were collected between April and September 2020 at MHH during the SARS CoV‐2 D614G wildtype infection waves, but before the Alpha, Delta, Beta and Omicron waves. Of the *n* = 25 ICU patients, *n* = 12 received Remdesivir. The pre‐pandemic, unexposed control sera were collected before May 2020 [13].

### 4.2 Study Approval

The study was approved by the MHH Ethics Committee. All patients or participants provided written informed consent before participation in the study (9001 BO K, 968–2011).

### 4.3 Multiplex Assays

Luminex‐based multiplex assays were used to quantify cytokines, chemokines, endothelial factors, SARS‐CoV‐2 S1‐, RBD‐ S2‐, and N‐specific IgG as well as autoantibodies. Plasma proteins were measured using Bio‐Plex ProTM Human Assays (Bio‐Rad, Hercules, USA), (12007283, 171B6009M, 171B6022M) as well as Milliplex MAP kit human cardiovascular disease panel 5 (Merck‐Millipore, Darmstadt, Germany, HCVD5MAG‐67K) following manufacturer's instructions in 1+1 dilutions. Autoantibodies were quantified using Milliplex MAP Human Cytokine Autoantibody Panel (HCYTAAB‐17K) and Milliplex MAP Human Autoimmune Autoantibody Panel (HAIAB‐10K; both Merck‐Millipore) according to manufacturer's instructions, plasma samples were diluted 1:100. SARS‐CoV‐2 S1‐, RBD‐, S2‐ and N‐specific IgG was detected using the SARS‐CoV‐2 Antigen Panel 1 IgG assay (Merck‐Millipore, HC19SERG1‐85K‐04) following manufacturer's instructions with 1:200 diluted plasma. MFI was used as readout.

### 4.4 Electrochemiluminescence (ECL) Multiplex Assays for Interference of ACE2 Binding to VOC Spike Domains

As a surrogate neutralisation assay [32, 33], multiplex assays V‐PLEX SARS‐CoV‐2 Panel 23 (IgG, K15567U, Meso Scale, Rockville, USA) and V‐PLEX SARS‐CoV‐2 Panel 28 (IgG, K15614, Mesoscale, USA) were used, measuring the capability of plasma antibodies to interfere with the binding of VOC spike‐protein or RBD to soluble human ACE2 receptor. SARS‐CoV‐2 VOC‐specific antibody inhibitory capacity (AIC) in plasma (1:100 diluted) was measured simultaneously against eight spike proteins from WT, Alpha, B.1.351 (Beta), P.1 (Gamma), AY.3 (Delta), AY.4 (Delta), AY.4.2 (Delta), and BA.1 (Omicron) variants and four RBD domains of BA.2 (Omicron), BA.2.12.2 (Omicron), BA.3 (Omicron), and BA.4/5 (Omicron). Samples were acquired by MESO QuickPlex SQ 120. AIC was calculated as % inhibition = [1 – average sample ECL/ average assay buffer ECL] × 100.

### 4.5 Flow Cytometry of PBMC

Flow cytometry was performed as recommended by the guidelines of EJI [34]. 100 µL whole blood EDTA samples were incubated with antibodies for surface staining in FACS Buffer (0.1% NaN_3_, 2.5% FCS in PBS) at 4°C for 30 min, followed by 15 min erythrocyte lysis using 1× BD lysing solution. Before acquisition, cells were washed with PBS. All antibodies used for FACS are listed in Table S1. Cells were acquired and analysed on an LSRII flow cytometer (BD Biosciences, Franklin Lakes, USA) using FACSDiva software (v8.0).

### 4.6 Quantification of Cells From EDTA Blood via TruCount Analyses

Absolute cell counts of lymphocytes in blood were calculated using BD TruCount tubes (BD Biosciences), following the manufacturer's instructions using 50 µL of whole blood. Samples were immediately acquired at the LSRII flow cytometer, and immune cell subsets were gated using FACSDiva Software. The absolute cell numbers (cell/µL) were determined by comparing cellular events to bead events. The number of cellular events was divided by the number of bead events and multiplied by the TruCount bead concentration.

### 4.7 Statistical Analyses

Statistical analyses of the data were performed with GraphPad Prism v9.0 software or with R version 4.4.3. The normality of the data was assessed using the Shapiro–Wilk Test (R base stats package). Based on the data distribution, parametric or non‐parametric tests were applied as appropriate. For non‐normally distributed data and factorial study designs, Aligned Rank Transform ANOVA (R package ARTool) was used, which allows factorial ANOVA and mixed‐model analyses including interaction terms without assuming normality. Post hoc pairwise comparisons were adjusted using the false discovery rate (FDR) method. For normally distributed data, multigroup comparisons were performed using linear mixed‐effects models (R packages lme4 and lmerTest). Pairwise comparisons were conducted using Tukey's test with Kenward–Roger correction (package emmeans). To account for repeated measurements, patient pseudo‐ID was included as a random effect. Both Aligned Rank Transform ANOVA and linear mixed‐effects model approaches are suitable for unequal sampling across groups, with UE and ICU sampled at a single time point, and LC sampled longitudinally.

Correlation analyses were performed using Spearman's rank‐order correlation test and shown as correlation matrices using the R packages corrplot and Hmisc or psych. Results were considered significant if p < 0.05. The statistical tests used in each analysis are indicated in the figure legends. All statistical tests, detailed model outputs, and R code are provided as an HTML file in the Supporting Information Materials.

### 4.8 Unsupervised Cluster and Principal Component Analyses

The cytokine/chemokine protein dataset was analysed using Qlucore Omics Explorer 3.7 (Lund, Sweden). Data were log_2_ transformed, scaled to mean zero, variance one. Prior to log_2_ transformation, values ≤ 0.01 were set to threshold = 0.01 to prevent zero/near‐zero values from disproportionately affecting variance estimates. Discriminating variables were determined using linear models, and multigroup analysis of variance (ANOVA) comparison, hierarchical clustering, and PCA were performed. Multiple comparisons were adjusted using the Benjamin–Hochberg method as implemented in Qlucore Omics Explorer software.

## Author Contributions

L.R. performed flow cytometry experiments as well as Luminex‐based and electrochemiluminescence‐based multiplex assays, analysed data, prepared figures, and wrote the manuscript. I.P. collected clinical data, provided patient samples, analysed data and helped write the manuscript. E.C. performed statistical analyses, prepared figures and helped write the manuscript. K.B. and J.K. performed Luminex‐based and electrochemiluminescence‐based multiplex assays. A.L.U. performed flow cytometry experiments and analysed data. N.D. collected clinical data and provided patient samples. A.S., L.B., J.S., T.W., and M.M.H. provided patient samples. J.F.K. supervised the study, performed experiments and wrote the manuscript. C.S.F. supervised and designed the study and wrote the manuscript. All authors contributed to the article and approved the submitted version.

## Ethics Statement

All participating subjects provided written informed consent, and the ethics committee of Hannover Medical School approved the study (no. 9001 BO K, 968–2011).

## Conflicts of Interest

Marius M. Hoeper has received fees for lectures and/or consultations from Acceleron, Actelion, Aerovate, Altavant, AOP, Bayer, Ferrer, GosamerBIO, Janssen, Keros, and MSD. The remaining authors declare no conflicts of interest.

## Supporting information




**Supporting File**: eji70169‐sup‐0001‐SupMat.pdf.

## Data Availability

The datasets used and/or analysed to support the findings of this study are available in this paper or the Supporting Information. Any other raw data that support the findings of this study are available from the corresponding author upon reasonable request.
