# Supplementary material for: Post‐Acute Sequelae Patients with Severe COVID‐19 History Show a Prolonged Inflammatory, Vascular Injury Pattern
Source: Eur J Immunol. 2026 Mar 29;56(4):e70169. doi: 10.1002/eji.70169 (PMC13033956; doi:10.1002/eji.70169)
Supplement: Supplementary file 1 — Supporting File: eji70169‐sup‐0001‐SupMat.pdf. [file EJI-56-e70169-s001.pdf]

Supporting Information

Post acute sequelae patients with severe COVID-19 history show a prolonged inflammatory, vascular injury pattern

Louisa Ruhl[1*], Isabell Pink[2*], Evgeny Chichelnitskiy[1*], Nora Drick[2], Andrea Sauer[2], Lennart Boblitz[2], Kerstin Beushausen[1], Jana Keil[1], Anna-Lena Ullrich[1], Julius Schmidt[3], Marius M. Hoeper[2,4], Tobias Welte[2,4†], Jenny F. Kühne[1‡], Christine S. Falk[1,4,5‡]

Correspondence:

Christine S. Falk, PhD, Institute of Transplant Immunology, Hannover Medical School, Hannover, Germany; falk.christine@mh-hannover.de

Supplementary Figure 1: Schematic illustration of blood sampling and antibody inhibitory capacity (AIC) against the spike-protein of SARS-CoV-2 variants.

Supplementary Figure 2: Flow cytometry gating strategies.

Supplementary Figure 3: Association between autoantibody prevalence and clinical parameters for Long-COVID.

Supplementary Figure 4: Altered immune cell phenotype in PASC patients.

Supplementary Figure 5: Frequencies of NK and Treg subsets.

Supplementary Figure 6: Age distribution of T cell subsets.

Supplementary Figure 7: Altered plasma protein signature of PASC patients.

Supplementary Figure 8: Altered plasma protein signature but no differences in clinical parameters or underlying diseases for the three LC patient subgroups.

Supplementary Figure 9: Plasma protein levels in UE and LC patients with obesity.

Supplementary Figure 10: Figure S10 *In vitro* stimulation of primary endo- and epithelial cells.

Supplementary Table 1: List of fluorescently labelled antibodies used for cell surface staining for flow cytometric analyses.

Supplementary Methods: Stimulation of primary human lung cells; RNA isolation and real-time PCR; Statistic codes and output

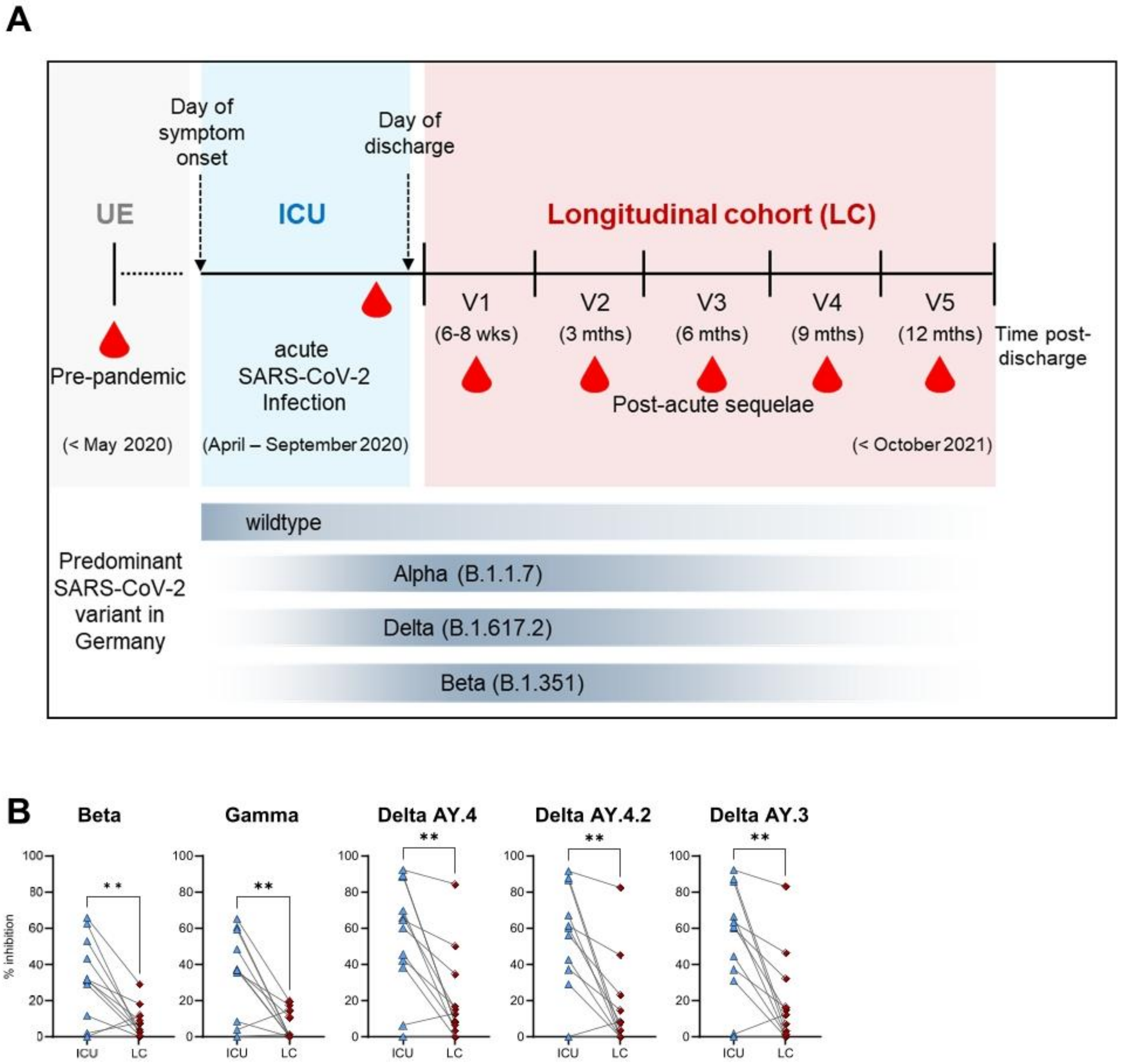

Figure S1 Schematic illustration of blood sampling and antibody inhibitory capacity (AIC) against the spike-protein of SARS-CoV-2 variants. (A) Scheme depicting the three cohorts analysed in this study, their respective blood sampling as well as the predominant SARS-CoV-2 variant in Germany at that time. **(B)** AIC was analysed using electrochemiluminescence-based multiplex assays and is displayed as % inhibition. AIC against VOCs (Beta, Gamma, Delta AY.3, Delta AY.4, Delta AY4.2) compared between matched samples from acute COVID-19 ICU patients which subsequently developed Long-COVID (n=12). Samples from LC were collected 6 months after hospital discharge.

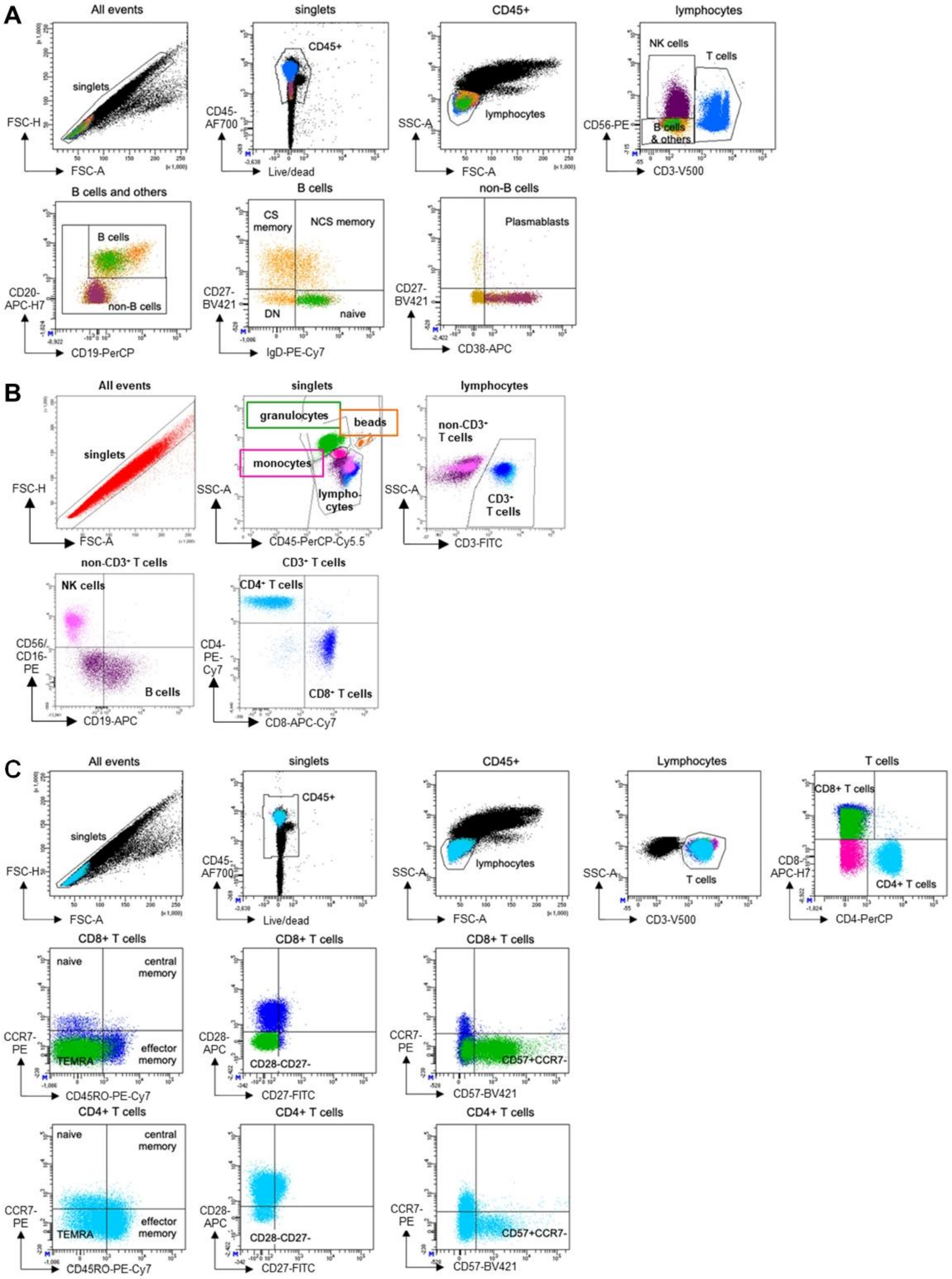

Figure S2 Flow cytometry gating strategies. Representative flow cytometry plots visualizing the gating strategy for **(A)** B cell subsets: naive (IgD^+CD27^-), double negative (DN, IgD^-CD27^-),

class-switched memory (CS memory, IgD^{-}CD27^{+}), non-class-switched memory (IgD^{+}CD27^{+}) and Plasmablasts (CD19^{-}CD27^{+}CD38^{+}). **(B)** Absolute lymphocyte cell counts (TruCount): CD4^{+} T cells (CD45^{+}CD3^{+}CD4^{+}), CD8^{+} T cells (CD45^{+}CD3^{+}CD8^{+}), NK cells (CD45^{+}CD3^{-}CD56/CD16^{+}), B cells (CD45^{+}CD3^{-}CD56/CD16^{-}CD19^{+}). **(C)** T cell subsets**:** naive (CCR7^{+}CD45RO^{-}), central memory (CM, CCR7^{+}CD45RO^{+}), effector memory (EM, CCR7^{-}CD45RO^{+}) and TEMRA (CCR7^{-}CD45RO^{-}).

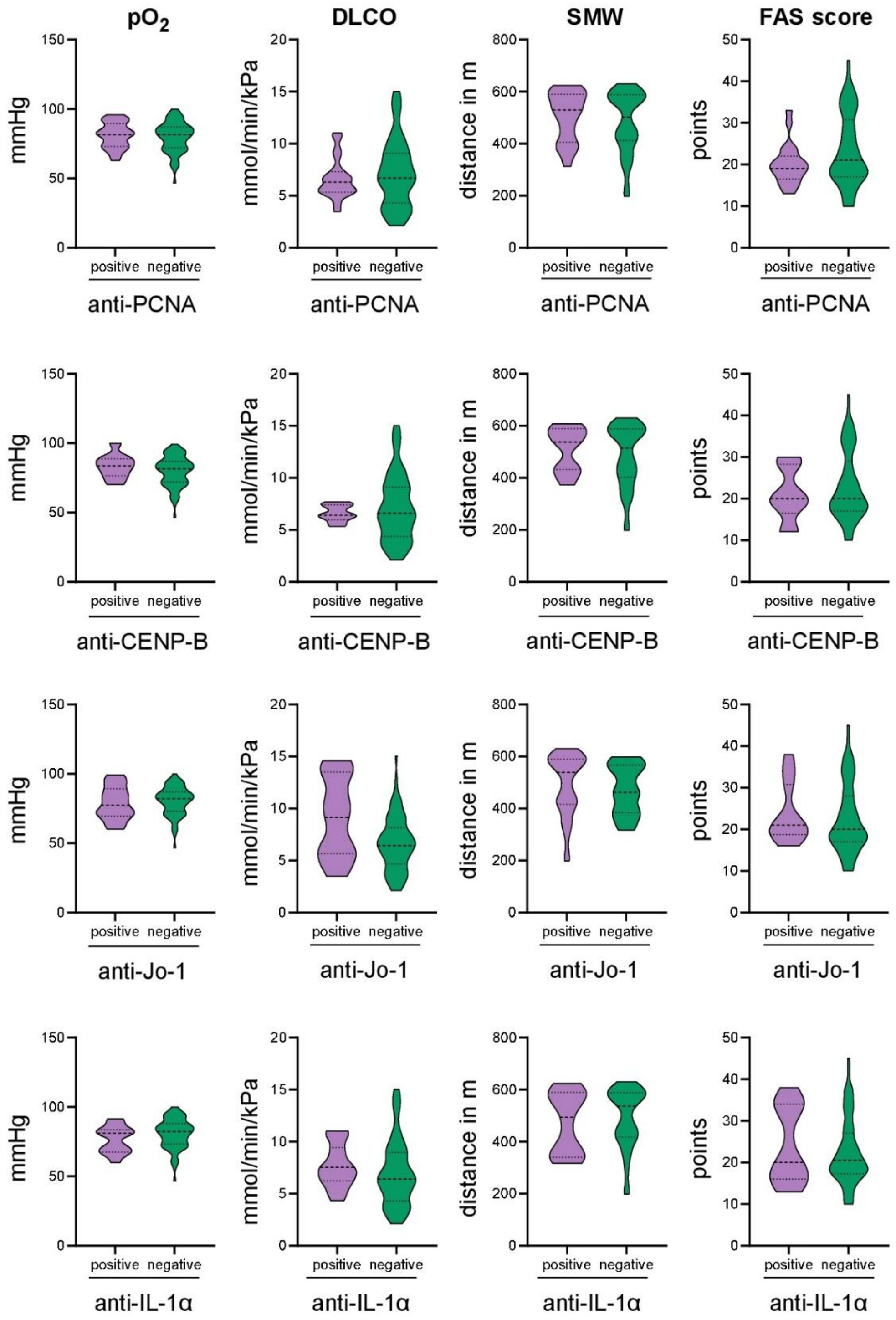

Figure S3 Association between autoantibody prevalence and clinical parameters for Long-COVID. Clinical parameters for Long-COVID in Long-COVID patients with (positive) or without (negative) anti-PCNA, anti-CENP-B, anti-Jo-1 or anti-IL-1α autoantibodies. Autoantibodies were measured by Luminex-based multiplex assay. pO_2: partial pressure of

oxygen, DLCO: Diffusing capacity of the lungs for carbon monoxide, SMW: six-minutes walking test, FAS score: fatigue score. Statistical analysis: t-test or Mann-Whitney test.

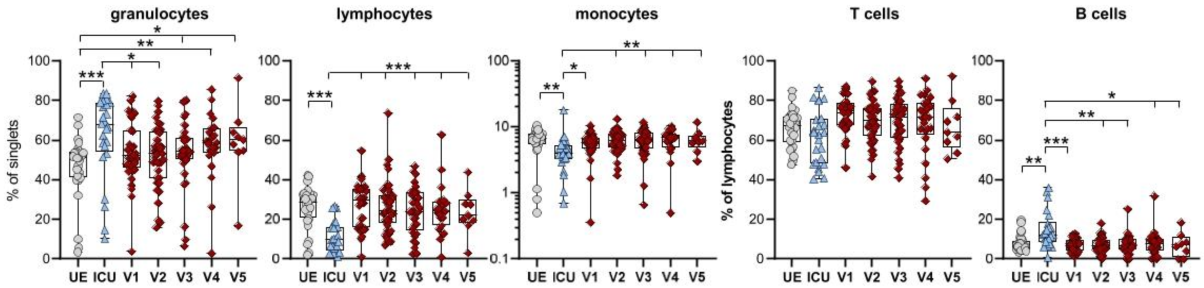

Figure S4 Altered immune cell phenotype in PASC patients. Immune cell composition was analysed by flow cytometry in peripheral blood. Immune cell frequencies from UE (n=28), ICU (n=25, last visit) and LC (n=139 samples in total, derived from 5 visits (V1-V5)).
Statistical analysis: Multigroup comparisons (Aligned Rank Transform ANOVA (ARTool) with FDR-adjusted post hoc testing for non-parametric data), or linear mixed-effects models with Kenward–Roger correction and Tukey's post hoc test for parametric data. Patient ID was included as a random effect to account for repeated measurements. *p < 0.05, **p < 0.01, ***p < 0.001.

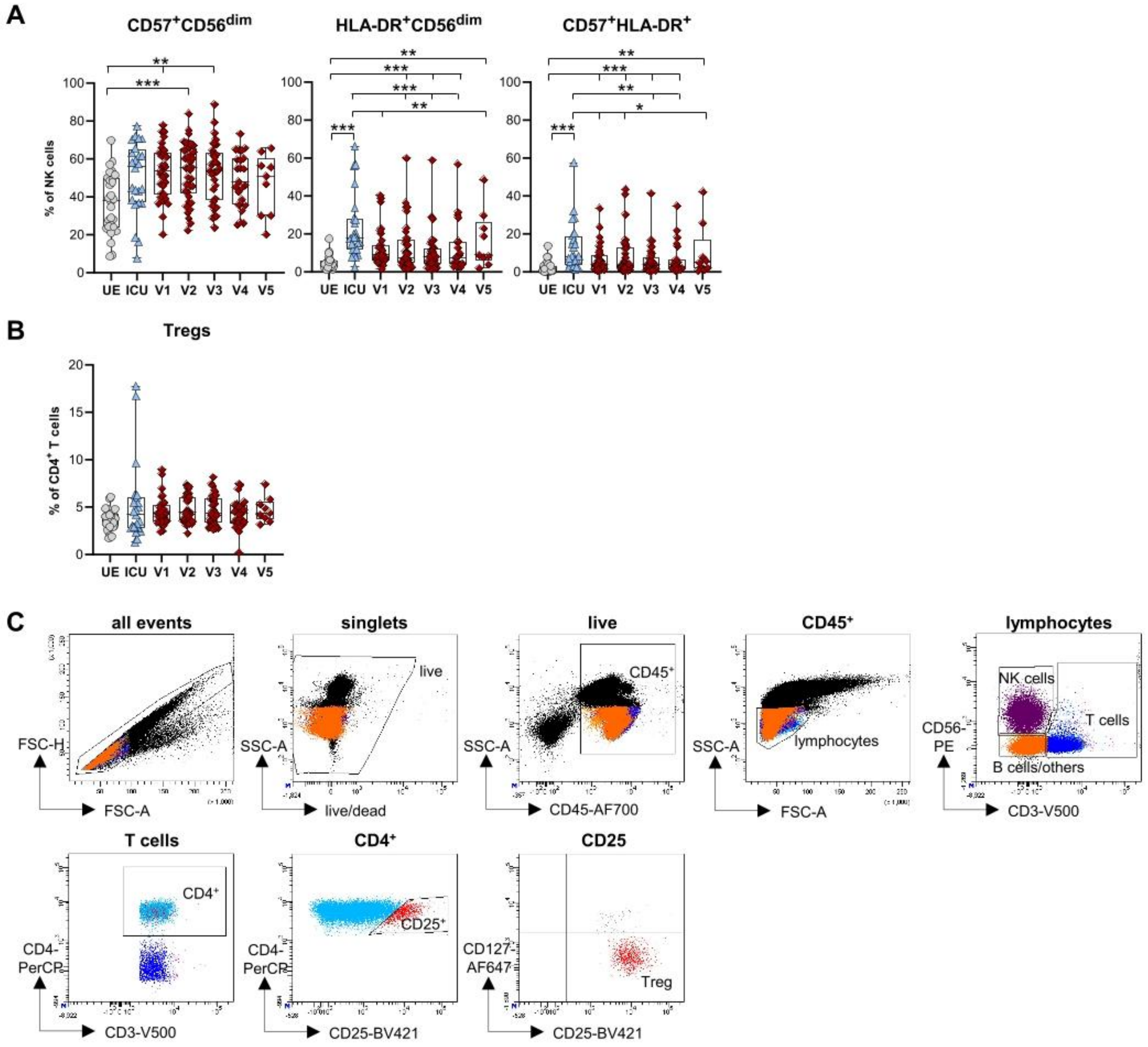

Figure S5 Frequencies of NK and Treg subsets. Immune cell frequencies were measured using flow cytometry. **(A)** NK cell subsets, **(B)** Tregs and **(C)** Treg gating strategy ($CD3^{+}CD4^{+}CD25^{+}CD127^{low/neg}$).

Treg: regulatory T cells, UE: unexposed donors (n=28), ICU: COVID-19 ICU patients (n=25, last visit), LC: longitudinal COVID-19 cohort (n=139 samples in total, derived from 5 visits (V1-V5)). Statistical analysis Statistical analysis: Multigroup comparisons (Aligned Rank Transform ANOVA (ARTool) with FDR-adjusted post hoc testing for non-parametric data), or linear mixed-effects models with Kenward–Roger correction and Tukey's post hoc test for parametric data. Patient ID was included as a random effect to account for repeated measurements. *p < 0.05, **p < 0.01, ***p < 0.001.

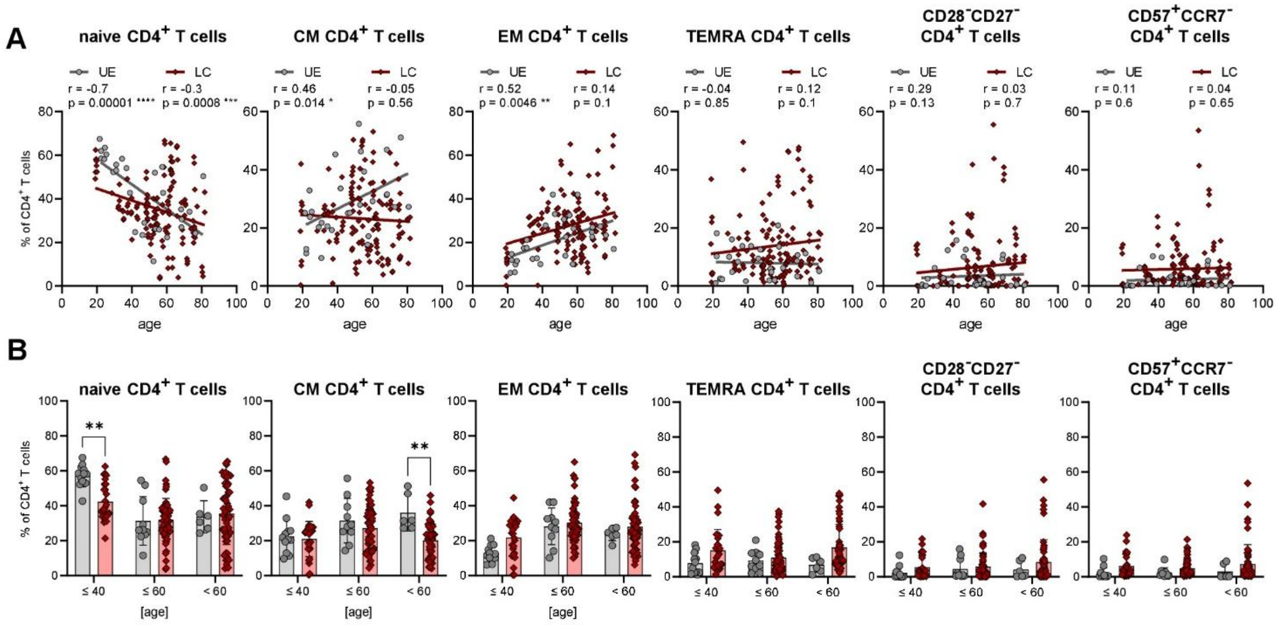

Figure S6 Age distribution of T cell subsets. Immune cell frequencies were measured using flow cytometry. **(A)** Spearman-correlation analysis between proportions of different T cell subsets and age from LC (n=139) and UE (n=25) with linear regression. **(B)** Comparison of immune cell frequencies between different age groups of LC and UE. UE are displayed in grey, LC are displayed in red. UE: ≤40 years n=12, 40-60 years n=9, >60 years n=7, LC: ≤40 years n=25, 40-60 years n=54, >60 years n=60.

UE: unexposed donors, LC: Long-COVID patients. Statistical analysis: (A) 2way ANOVA, (B) Spearman rank correlation. **p < 0.01.

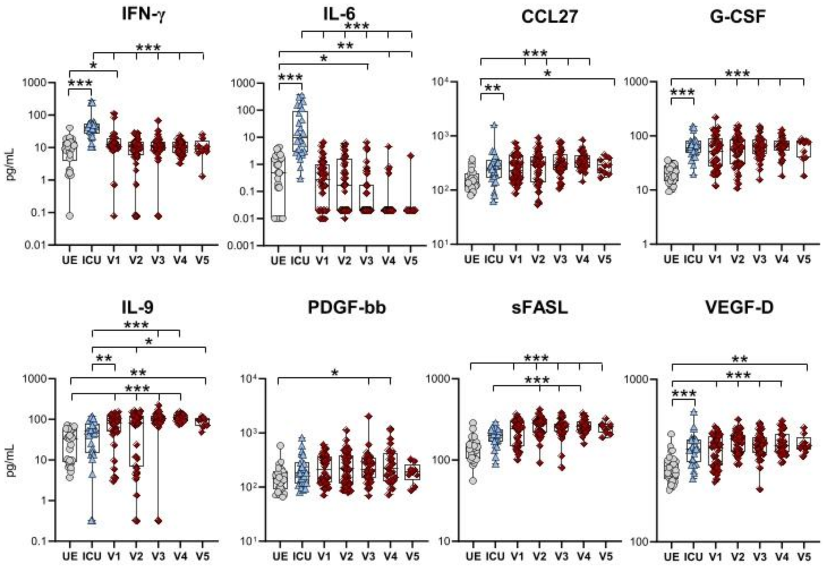

Figure S7 Altered plasma protein signature of PASC patients. Plasma protein concentrations in patient plasma were measured by Luminex-based multiplex assay.
UE: unexposed donors (n=28), ICU: COVID-19 ICU patients (n=25, last visit), LC: longitudinal COVID-19 cohort (n=139 samples in total, derived from 5 visits (V1-V5)).
Statistical analysis Statistical analysis: Multigroup comparisons (Aligned Rank Transform ANOVA (ARTool) with FDR-adjusted post hoc testing for non-parametric data), or linear mixed-effects models with Kenward–Roger correction and Tukey's post hoc test for parametric data. Patient ID was included as a random effect to account for repeated measurements. *p < 0.05, **p < 0.01, ***p < 0.001, ****p < 0.0001.

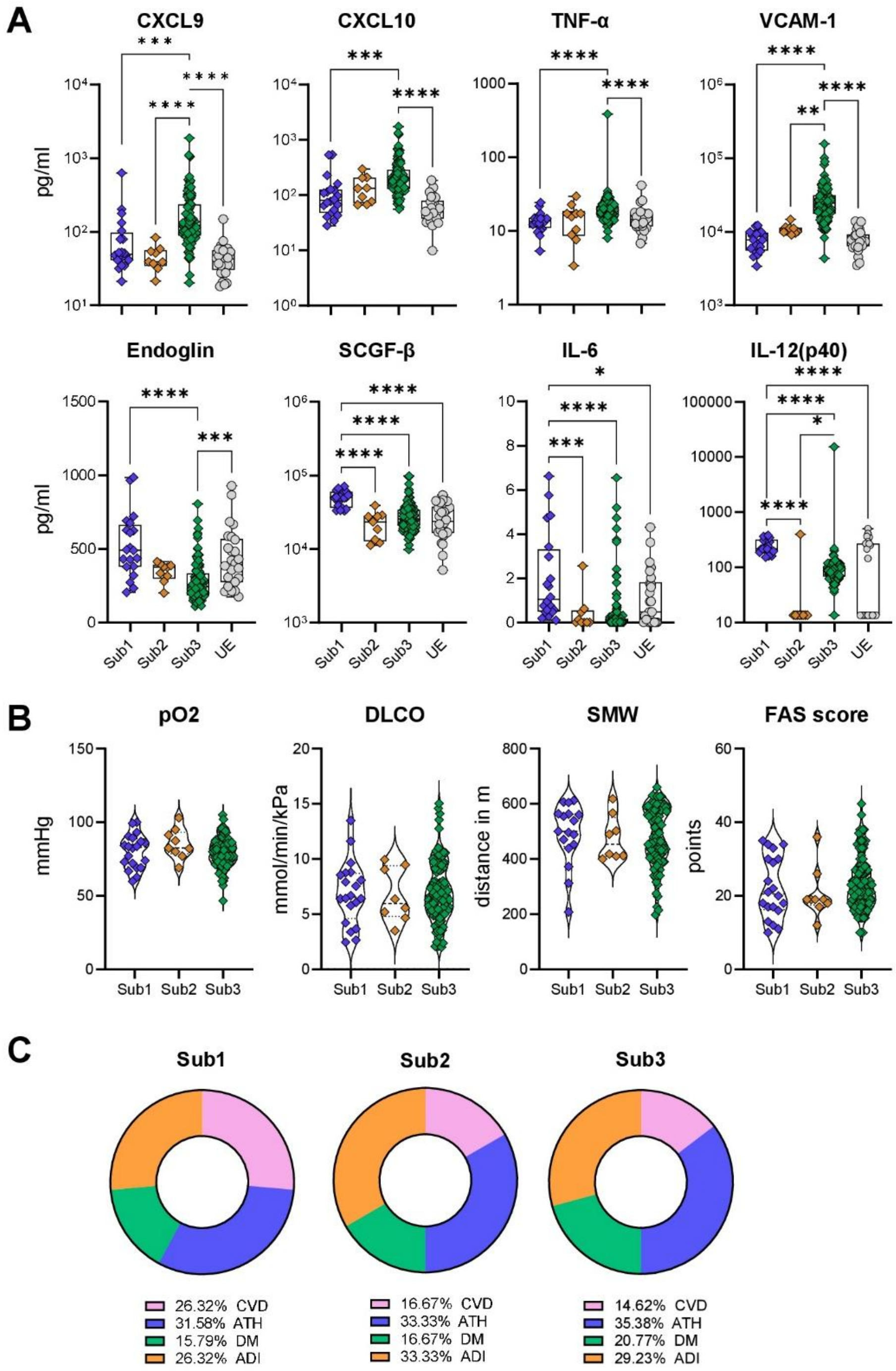

Figure S8 Altered plasma protein signature but no differences in clinical parameters or underlying diseases for the three LC patient subgroups. (A) Plasma protein concentrations in patient plasma were measured by Luminex-based multiplex assay. Plasma protein levels in UE (n=29) and LC subgroups. Sub1 n=20, Sub2 n=10, Sub3 n=109. **(B)** Clinical parameters for LC subgroups as identified in Figure 4D. **(C)** Donut chart for Sub1 to Sub3 displaying underlying diseases (in %). Sub1 n=20, Sub2 n=10, Sub3 n=109. Kruskal-Wallis test with Dunn's multiple comparison test. *p < 0.05, **p < 0.01, ***p < 0.001, ****p < 0.0001.

pO_2: partial pressure of oxygen, DLCO: Diffusing capacity of the lungs for carbon monoxide, SMW: six-minutes walking test, FAS score: fatigue score. UE: unexposed donors.

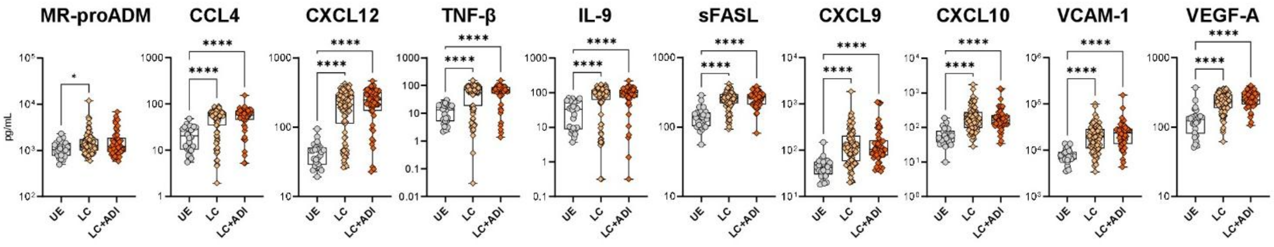

Figure S9 Plasma protein levels in UE and LC patients with obesity. Plasma protein concentrations in patient plasma were measured by Luminex-based multiplex assay. LC were subdivided into Long-COVID patients (LC) without underlying obesity and Long-COVID patients with obesity (LC+ADI). UE (n=26), LC (n=87), and LC+ADI (n=50). Kruskal-Wallis test with Dunn's multiple comparison test. *$p < 0.05$, **$p < 0.01$, ***$p < 0.001$, ****$p < 0.0001$.

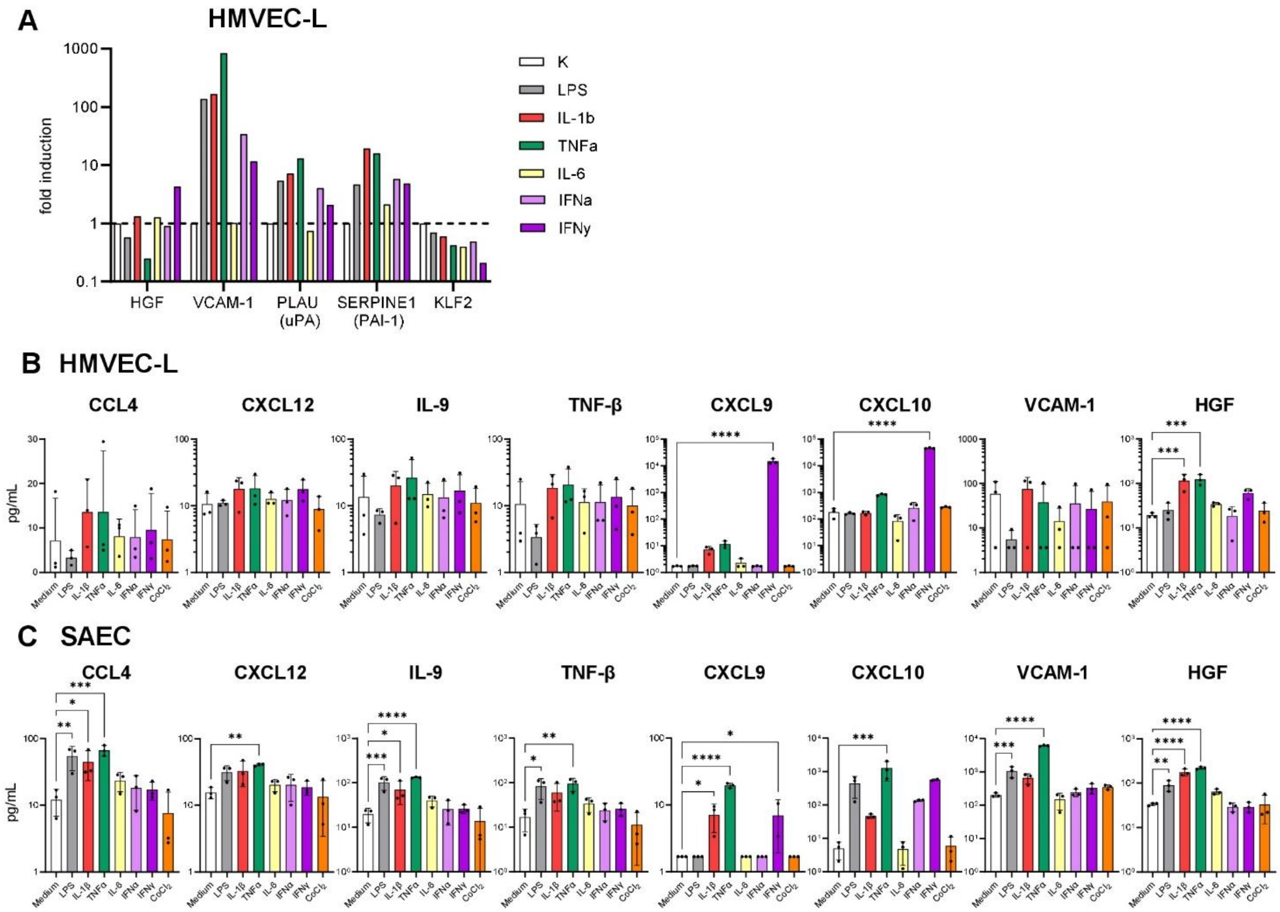

Figure S10 *In vitro* stimulation of primary endo- and epithelial cells. Stimulation of primary human lung cells, RNA isolation and real-time PCR were performed as described in Supplementary Methods (see below). **(A)** mRNA expression after stimulation of HMVEC-L with inflammatory mediators. mRNA expression is represented as fold induction compared to unstimulated control. **(B, C)** Concentrations of soluble factors like cytokines, chemokines, growth factors and endothelial factors from supernatant of stimulated **(B)** HMVEC-L or **(C)** SAEC. Soluble factors were measured via Luminex-based multiplex assays. HMVEC-L: human lung microvascular endothelial cells, SAEC: human small airway epithelial cells.
Statistical analysis: (B) ANOVA test with Dunnett's multiple comparison test or Kruskal–Wallis with test with Dunn's multiple comparison test were performed. *$p < 0.05$, **$p < 0.01$, ***$p < 0.001$, ****$p < 0.0001$.

Supplementary Table 1. List of fluorescently labelled antibodies used for cell surface staining for flow cytometric analyses.

Antigen	Flurochrome	Manufacturer
CD3	V500	BD Bioscience
CD3	APC-H7	BD Bioscience
CD3	PerCP	BD Bioscience
CD4	PerCP	BD Bioscience
CD6	FITC	BD Bioscience
CD8	APC-H7	BD Bioscience
CD14	PE-Cy7	BD Bioscience
CD16	APC	BD Bioscience
CD19	PerCP	BD Bioscience
CD20	APC-H7	BD Bioscience
CD24	FITC	BD Bioscience
CD25	BV421	BD Bioscience
CD27	FITC	BD Bioscience
CD27	BV421	BD Bioscience
CD28	APC	BD Bioscience
CD38	APC	BD Bioscience
CD45	AF700	Biolegend
CD45	APC-H7	BD Bioscience
CD45R0	PE-Cy7	BD Bioscience
CD56	PE	BD Bioscience
CD57	BV421	BD Bioscience
CD69	FITC	BD Bioscience
CD127	AF647	BD Bioscience
CD197 (CCR7)	PE	BD Bioscience
HLA-DR	V500	BD Bioscience
IgD	PE-Cy7	BD Bioscience

Supplementary Methods

Stimulation of primary human lung cells

Primary human lung cells, i.e. microvascular endothelial cells (HMVEC-L) and small airway epithelial cells (SAEC) were stimulated *in vitro* with cytokines or $CoCl_2$ to mimic hypoxia. Triplicates of $2x10^4$ SAEC and $1,5x10^4$ HMVEC-L cells were stimulated with LPS 0.5 µg/ml; IL-1β 10 ng/ml; TNF-α 20 ng/ml; IL-6 50 ng/ml; IFN-α 100 U/ml; IFN-γ 100 U/ml; $CoCl_2$ 100 µM and RPMI1640 medium (5% FBS) as control for 48h at 37°C and 5% CO_2, supernatant was frozen at -20°C.

RNA isolation and real-time PCR

Total RNA was isolated from stimulated primary HMVEC-L or SAEC cells using EXTRAzol RNA isolation kit (DNA Gdansk, Blirt S.A., Danzig, Poland) according to manufacturer’s instructions. RNA was dissolved in RNAse-free H_2O and RNA concentration was determined using Nanodrop (ThermoFisher Scientific). 750 ng RNA was applied for cDNA synthesis using RevertAid H Minus First Strand cDNA Synthesis Kit (ThermoFisher Scientific, Waltham, USA). Real time polymerase chase reaction (RT-PCR) was performed with LightCycler96 (Roche, Basel, Switzerland) utilizing FastStart essential DNA probes Master (Roche) and Taqman Gene Expression Assays (ThermoFisher Scientific) for the following genes: Glyceraldehyde-3-phosphate dehydrogenase (GAPDH, Hs99999905_m1), IL-10 (Hs00173499_m1) and IFN-γ, Hs0098921_m1). Target mRNA expression is presented as fold induction ($2^{-\Delta\Delta Ct}$) compared to unstimulated samples at time point *pre* and the reference gene GAPDH. Data were acquired and analysed using LightCycler Software Version 1.1.0.1320 (Roche).

Statistic codes and output

All statistical tests, detailed model outputs, and R code can be assessed via: Revision Ruhl et al EJI 2025
